# Glycolytic System in Axons Supplement Decreased ATP Levels after Axotomy of the Peripheral Nerve

**DOI:** 10.1523/ENEURO.0353-22.2023

**Published:** 2023-03-17

**Authors:** Tomofumi Takenaka, Yuichiro Ohnishi, Masamichi Yamamoto, Daiki Setoyama, Haruhiko Kishima

**Affiliations:** 1 Department of neurosurgery, Graduate School of Medicine, Osaka University, Osaka, 565-0871, Japan; 2 Department of Research Promotion and Management, National Cerebral and Cardiovascular Center, Osaka, 564-8565, Japan; 3 Department of Neurosurgery, Osaka Gyoumeikan Hospital, Osaka, 554-0012, Japan; 4 Department of Clinical Chemistry and Laboratory Medicine, Graduate School of Medical Sciences, Kyushu University, Fukuoka, 812-8582, Japan

**Keywords:** ATP, glycolytic system, *in vivo* imaging, mitochondria, Wallerian degeneration

## Abstract

Wallerian degeneration (WD) occurs in the early stages of numerous neurologic disorders, and clarifying WD pathology is crucial for the advancement of neurologic therapies. ATP is acknowledged as one of the key pathologic substances in WD. The ATP-related pathologic pathways that regulate WD have been defined. The elevation of ATP levels in axon contributes to delay WD and protects axons. However, ATP is necessary for the active processes to proceed WD, given that WD is stringently managed by auto-destruction programs. But little is known about the bioenergetics during WD. In this study, we made sciatic nerve transection models for *GO-ATeam2* knock-in rats and mice. We presented the spatiotemporal ATP distribution in the injured axons with *in vivo* ATP imaging systems, and investigated the metabolic source of ATP in the distal nerve stump. A gradual decrease in ATP levels was observed before the progression of WD. In addition, the glycolytic system and monocarboxylate transporters (MCTs) were activated in Schwann cells following axotomy. Interestingly, in axons, we found the activation of glycolytic system and the inactivation of the tricarboxylic acid (TCA) cycle. Glycolytic inhibitors, 2-deoxyglucose (2-DG) and MCT inhibitors, a-cyano-4-hydroxycinnamic acid (4-CIN) decreased ATP and enhanced WD progression, whereas mitochondrial pyruvate carrier (MPC) inhibitors (MSDC-0160) did not change. Finally, ethyl pyruvate (EP) increased ATP levels and delayed WD. Together, our findings suggest that glycolytic system, both in Schwann cells and axons, is the main source of maintaining ATP levels in the distal nerve stump.

## Significance Statement

Wallerian degeneration (WD) after axotomy is associated with decreasing ATP levels. To maintain ATP levels, Schwann cells activate the glycolytic systems and produce monocarboxylate, which is transported to axons via monocarboxylate transporters (MCTs). Axons also activate the glycolysis system to obtain ATP, and inactivate tricarboxylic acid (TCA) cycle because of mitochondrial degeneration. The glycolysis with MCT-induced monocarboxylate transport contributes to the ATP production in degenerative axons.

## Introduction

Wallerian degeneration (WD) refers to programmed axon degeneration, which is classically defined as the pathology of the distal nerve stump following injury (Waller, 1851). Recent studies revealed that key pathologic features of WD occur in chronic neurologic disorders as well as acute traumatic injuries (J.T. Wang et al., 2012; Conforti et al., 2014; Coleman and Höke, 2020), to the mammalian CNS or peripheral nervous system (PNS; Vargas and Barres, 2007; Beirowski et al., 2010). Therefore, elucidating the pathology of WD is important in developing therapies that can prevent or delay axonal degeneration and loss of function in various axonopathy-driven disorders, such as peripheral and central nerve injury, cerebral infarction, and neurologic disorders.

ATP is one of the key pathologic substances in WD. The elevation of ATP levels in axons delays WD (J. Wang et al., 2005; Yang et al., 2015), and supports axon regeneration (Han et al., 2016; Zhou et al., 2016). Previous studies have been reported the ATP-related pathologic pathways involved in WD. First, ATP is used in the salvage pathway. Nicotinamide mononucleotide (NMN) and ATP are catalyzed by nicotinamide mononucleotide adenylyl transferase 2 (NMNAT2) to form nicotinamide adenine dinucleotide (NAD^+^; Lau et al., 2009; Gilley and Coleman, 2010). NAD^+^ is a critical factor in maintaining the distal nerve stump ATP levels during WD (Araki et al., 2004; Gerdts et al., 2015). Second, ATP is required for WD progression (Conforti et al., 2014). One of the main proposed mechanisms is axonal autophagy (Nixon, 2013), which is thought to contribute to local ATP production that drives WD (Wakatsuki et al., 2017). However, from a bioenergetics perspective, the specifics of these salvage pathways and auto-destruction programs in WD remains unknown (J.T. Wang et al., 2012; Yang et al., 2015; Gerdts et al., 2016; Babetto et al., 2020; Coleman and Höke, 2020). Especially, the spatiotemporal distribution and the metabolic source of ATP during WD remains elusive.

Recent studies suggested that activating the glycolytic system in Schwann cells and monocarboxylate supplementation to axons via monocarboxylate transporters (MCTs) is important for maintaining ATP levels, delaying WD, and promoting axon regeneration (Babetto et al., 2020; Li et al., 2020; Trimarco and Taveggia, 2020). The metabolic interaction between Schwann cells and axons is important for neuronal survival (Bouçanova and Chrast, 2020). However, mitochondria become dysfunctional in the early stages of WD (B. Wang et al., 2021), with dilated and disorganized cristae (J. Wang et al., 2011), and mitochondrial dysfunction would lead to reduced ATP production in the tricarboxylic acid (TCA) cycle (Pivovarova and Andrews, 2010); therefore, it is questionable whether axonal mitochondria metabolize the transported monocarboxylates in degenerative axons. Furthermore, axons in the distal nerve stump are incapable of transporting mitochondria to the caudal side, which results in a dramatic decline in both anterograde and retrograde axonal transport 24 h after axotomy (Misgeld et al., 2007). Study results remain controversial and the ATP-generating metabolic pathways, using monocarboxylates, in injured axons remain undefined.

In this study, we investigated the spatiotemporal ATP levels in injured peripheral nerves by using *in vivo* ATP imaging systems (Nakano et al., 2011; Yamamoto et al., 2019; Ohnishi et al., 2021; Nakajima et al., 2022). In addition, we explored the metabolic source of ATP in degenerative axons using mass spectrometry, histologic analysis, and pharmacological intervention.

## Materials and Methods

### Animals

All procedures were performed in accordance with the guidelines of the Laboratory Animal Care and Use Committee of Osaka University, Japan (No. 29-025-015). *GO-ATeam2* knock-in mice and rats were generated by M.Y. Briefly, we employed a knock-in strategy targeting the Rosa26 locus and the CAG promoter to regulate transcription, for generating both *GO-ATeam2* knock-in mice (Yamamoto et al., 2019) and rats (our unpublished observations). *GO-ATeam2* knock-in mice were generated by inducing targeting vectors into G4 ES cells through electroporation and breeding chimeric mice for at least five generations. *GO-ATeam2* knock-in rats were generated by CRISPR/Cas9-based genome editing system (Cong et al., 2013). Mice and rats were housed in cages of three animals at 24 ± 2°C under a 12/12 h light/dark cycle (8 P.M. lights off, 8 A.M. lights on), and were allowed free access to fast water overnight. The cages were cleaned twice a week. *GO-ATeam2* knock-in rats (SD background, 300–450 g, four to six rats per group) were used to measure ATP levels by fluorescence microscopy and histologic evaluation. *GO-ATeam2* knock-in mice (ICR background, 35–45 g, three to six mice per group) were used to measure ATP levels using fluorescence microscopy or two-photon microscopy, histologic evaluation, mass spectrometry, and local administration of inhibitor or ethyl pyruvate (EP). In the earlier part of the experiment, we used rats, which are more suitable for modeling human disorders and easier to surgically manipulate (Adalbert et al., 2005). As the experiment proceeds, we used mice because the experiments involving two-photon microscopy were unsuitable for the body size of rats. Mixed sexes were used because previous studies showed no significant difference between the sexes and the rates of axon degeneration because of injury (Babetto et al., 2020). All animals were 7–12 weeks in age. We made the best effort to minimize the number of animals used.

### Sciatic nerve transection models

We created the same transection model for GO-ATeam2 knock-in mice and rats as previously reported (Savastano et al., 2014). Briefly, mice and rats were deeply anesthetized with 4% isoflurane and maintained with 2% isoflurane in air. The animal’s body temperature was maintained at 36.0–37.0°C using a heating pad. A minimum skin incision was made 5 mm caudal and along the femur, and the right sciatic nerves were gently exposed and transected with micro scissors at the level of the sciatic notch. The wound was closed using surgical thread. During the target period, animals were killed by deep sedation after ATP imaging or harvesting of sciatic nerve lesions. Histologic evaluation (epifluorescence, confocal microscopy, electron microscopy) was performed 3 mm distal from the sectional end (Extended Data Figs. 1-1, 3-1). Distal sciatic nerve stumps (∼7-mm continuous from the sectional end) were harvested for mass spectrometry. Separate individuals were used for ATP imaging, histologic evaluation, and mass spectrometry.

### ATP imaging

**Figure 1.**
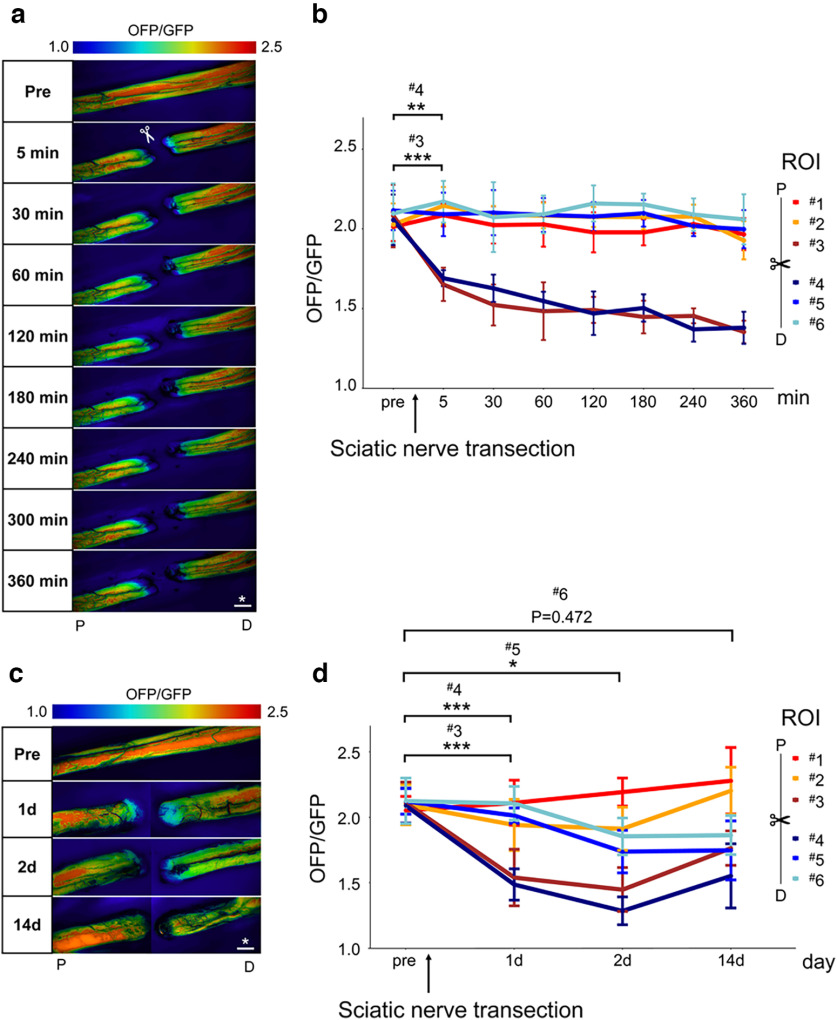
Spatiotemporal change for rat sciatic nerve whole ATP levels with fluorescence microscope after axotomy. ***a***, Representative whole nerve intracellular ATP images before (pre) and 5, 30, 60, 120, 180, 240, 300, and 360 min after axotomy in the same individuals. Scale bar, * 1 mm. P, proximal. D, distal. ***b***, The change of ATP levels with six ROIs (from ROI ^#^1 to ^#^6; *n* = 4 rat per group). Distal nerve stump ATP levels (ROI ^#^5 and ^#^6) were maintained over 360 min. ***c***, Representative whole nerve intracellular ATP images before (pre), and 1 d (24 h), 2 d (48 h), and 14 d after axotomy in the separate individuals. Scale bar, * 1 mm. P, proximal. D, distal. ***d***, The change of ATP levels with six ROIs (from ROI ^#^1 to ^#^6; *n* = 6 rat per group). Distal nerve stump ATP levels (ROI ^#^5 and ^#^6) were significantly decreased from 2 d after axotomy. The ROI for measuring ATP levels was set on six regions: three proximal (from ROI ^#^1 to ^#^3) and three distal (from ROI ^#^4 to ^#^6) sites for axotomy, and a 1-mm square with 500-μm spacing (Extended Data Fig. 1-1). The OFP/GFP ratios ranged from 1.0 to 2.5. Error bars indicate SD; *p < 0.05, **p < 0.01, ***p < 0.001, two-way ANOVA followed by the Tukey’s *post hoc* test. ROI, region of interest.

The *GO-ATeam2* probe expresses a fluorescence/Forster resonance energy transfer (FRET) pair as an ATP biosensor with orange fluorescent protein (OFP) and green fluorescent protein (GFP; Nakano et al., 2011). Specific binding of ATP to the ε subunit of the *GO-ATeam2* probe causes a conformational change in the ε subunit from the extended to the retracted form, increasing the FRET efficiency (Yagi, 2007). OFP and GFP were used as FRET donors and acceptors, respectively, and the OFP/GFP ratio was quantitatively calculated as the ATP levels in the cytoplasm. The obtained OFP/GFP ratio was applied to the following equation: FRET ratio = 1.52 × [ATP]^1.7/([ATP]^1.7 + 2.22) + 0.44, and then, ATP concentration was calculated. The coefficients were carefully determined based on two methods (Yamamoto et al., 2019). First, mouse embryonic fibroblasts were obtained from *GO-ATeam2* knock-in mice. After piercing the fibroblast membrane, we applied different concentrations of ATP, monitored the FRET ratio, and determined coefficients based on the function for fitting ATP concentration to the FRET ratio. Second, we performed a luciferase assay of ATP concentration (Tissue ATP assay kit; TOYO B-NET) in fertilized eggs from *GO-ATeam2* knock-in mice. Mice were injected with ATP synthetase inhibitors (2DG plus antimycin A), and the time course of the FRET ratio was monitored. Coefficients obtained by the two methods were substantially identical, indicating that they were appropriate for use in the present study. *In vivo* ATP imaging was performed using fluorescence microscopy and two-photon microscopy, as previously reported (Yamamoto et al., 2019; Ohnishi et al., 2021). Briefly, *GO-ATeam2* knock-in rats or mice were anesthetized with 2% isoflurane, and sciatic nerves were exposed and photographed under general anesthesia. A blackout curtain was placed around the nerve during photography to prevent the epineurium and fibrofatty tissue from interfering with ATP levels. Photographs were taken at designated times for each experiment. We used both GO-ATeam2 knock-in rats and mice for fluorescence microscopy (M165 FC; Leica Microsystems). An exciting light (470/40 nm, 2.5 W under objective lens) was applied, and a dichroic mirror 540 DCLP of 515/30 and 575/40 was used for GFP and OFP emission (DualView2 filter sets, INDEC Biosystems). The exposure time was 1000 ms. Fluorescence emission in the *GO-ATeam2* probe was captured using a CCD camera (ORCA-Flash 4.0, Hamamatsu Photonics KK Shizuoka), which could evaluate whole nerve ATP levels. We used only *GO-ATeam2* knock-in mice for two-photon microscopy (A1R MP+; Nikon) because the size of the mouse was suitable for imaging. Exciting light (920 nm, 20 W under objective lens) was applied, BP525/50 filters were used for emission, and DM560 and DM640 filters were used for excitation fluorescence separation. For the center of the longitudinal nerve cross-section of the sciatic nerve, fluorescence emission in the *GO-ATeam2* probe was captured. For fluorescence and two-photon microscopy, the imaging data were analyzed using MetaMorph software (Molecular Devices). For FRET signals at the chosen region of interest (ROI), OFP/GFP ratios were calculated with subtraction of the background signal to normalize the background condition. We set different ROI for each animal species or microscope. Six 1 × 1-mm square ROIs were chosen for fluorescence microscopy in rats, three proximal and three distal to the sectional end, with 500-μm spacing for each ROI (Extended Data Fig. 1-1). For fluorescence microscopy in mice, 3 mm distal from the sectional end, with a 500 × 500-μm square ROI, was chosen (Extended Data Fig. 7-2). For two-photon microscope experiments in mice, to avoid the interference of the epineurium or fibrofatty tissue unrelated to internal nerves, 3 mm distal from the sectional end, the center of the longitudinal nerve cross-section, with a 300 × 300-μm square ROI was chosen (Extended Data Fig. 3-1). Fluorescence microscopy imaging reflects whole nerve ATP levels, and two-photon microscopy imaging reflects the longitudinal nerve cross-section ATP levels. The same individuals were used for fluorescence microscopy until 120 min (Extended Data Fig. 7-1) or 360 min (Fig. 1*a*,*b*) after axotomy, and the separate individuals were used for the other experiments including fluorescence and two-photon microscopy. This was because of the restrictions on the length of time of guidelines of our institute that animals were allowed to be kept outside of the animal laboratory.

10.1523/ENEURO.0353-22.2023.f1-1 Extended Data Figure 1-1 Experimental system for ATP levels with fluorescence microscopy and histological analysis in rats. A schematic illustration of whole nerve ATP levels measurement for GO-ATeam2 rat. All of fluorescence emission in the GO-ATeam2 probe was captured with fluorescence microscopy. ATP levels were intermittently measured in six ROIs (from ROI ^#^1 to ^#^6), three proximal and three distal sites for axotomy, 1-mm square with 500-μm spacing in the same individual. OFP/GFP ratio ranges were from 1.0 to 2.5. The histological analysis was performed 3 mm distal from the sectional end, correspond to ROI ^#^6. Scale bar, * 1 mm,** 1-mm square ROI. P, proximal. D, distal. Download Figure 1-1, TIF file.

### Myelinated axon measurement

Tissue preparation was performed 3 mm distal to the sectional end for the injured nerves and contralateral uninjured nerves. Samples were fixed in 2.5% glutaraldehyde in 0.1 m cacodylate buffer (pH 7.4) overnight at 4°C, postfixed with 1% OsO4 and embedded in epoxy resin. Semi-thin cross-sections (1 μm) were stained with an aqueous solution containing 0.5% toluidine blue and scanned using an epifluorescent microscope (BZ-X700, Keyence). Ultrathin sections (90 nm) were scanned using a transmission electron microscope (H-7500; Hitachi). Histologic quantification was conducted using epifluorescent microscopy by a blinded investigator using established nonbiased counting methods (Beirowski et al., 2005; Babetto et al., 2013; Viader et al., 2013; Sasaki et al., 2018). Briefly, whole sciatic nerve semi-thin section was imaged at 100× magnification with tile scanning, and the total area of the nerve was measured (BZ-X700 Analyzer, Keyence). Then, four randomly selected images were imaged (108 × 144 μm) at 1000× magnification with using oil-immersion lens, and intact myelinated axons were counted (ImageJ; v1.52p, and Cell Counter Plugin) in each image. Finally, based on the total area and the number of intact myelinated axons measured in each image, the number of axons in the entire nerve was estimated. The survival criteria for intact axons were normal myelin sheaths, uniform axoplasm, and intact mitochondria, as per the electron microscope findings described below.

### G-ratio calculation, mitochondrial diameter, and atypicality

Ultrathin sciatic nerve cross-sections (90 nm) were analyzed for mitochondrial diameter or atypicality, axon diameter, and G-ratio (the ratio between the inner axon and outer myelin perimeter) by electron microscopy. For myelinated axons, the inner and outer diameters of the myelin sheath were measured from 100 fibers randomly selected from four individuals per group using the GRatio software plug-in, available online (http://gratio.efil.de), implemented in ImageJ (https://imagej.nih.gov/ij), which allowed semi-automatic G-ratio calculation (Beirowski et al., 2017; Roberts et al., 2017; Alvarez-Prats et al., 2018). The mean G-ratio was calculated for each individual. Fifty mitochondria from each of the four individuals were measured in cross-sections, and the mean mitochondrial diameter was calculated for each individual (Barrientos et al., 2011). We defined atypical mitochondria as having a swollen shape, vacuolization or cavitation of the matrix, and lack of continuity in the outer and inner membranes (Lenzi et al., 2012; Natale et al., 2015; Chen et al., 2017).

### Immunohistochemistry

The frozen section procedure was performed 3 mm distal to the injured nerves’ sectional end and the contralateral uninjured nerves. Sciatic nerves from mice or rats were fixed in 4% paraformaldehyde/0.1 m PBS overnight, sucrose gradient (10%, 20%, 30% in 1× PBS), embedded in Tissue-Tek O.C.T. compound (Tissue Tek, Sakura Finetek Japan Co, Ltd.), and sectioned at 10 μm on a cryostat (CM3050 S, Leica Microsystems). Samples were washed in 1× PBS for 20 min and then blocked with 1% ECL Prime Blocking Reagent in 0.1% Triton X-100 1× PBS for 1 h at room temperature. Sections were incubated in primary antibodies in blocking/permeabilized solution overnight at 4°C, washed in 1× PBS for 10 min, and incubated with secondary antibodies for 1 h at room temperature. Samples were washed twice in 1× PBS for 15 min and mounted in VECTASHIELD Antifade Mounting Medium containing DAPI (H-1200, Vector Laboratories). The following primary and secondary antibodies and these concentrations were used as follows: Mouse-Monoclonal Anti-NF-H, 1:1000, NBP1-05210, Novus Biologicals; Rabbit polyclonal antibody Anti-S-100B, 1:500, GTX129573, Genetex; Rat-Monoclonal Anti-CD-68, 1:100, 14-0681-82, Invitrogen; Mouse-Monoclonal Anti-HK1, 1:100, sc-46695, Santa Cruz Biotechnology Inc; Mouse-Monoclonal Anti-PFK1, 1:100, sc-377346, Santa Cruz Biotechnology Inc; Mouse-Monoclonal Anti-LDHA, 1:100, sc-137243, Santa Cruz Biotechnology Inc; Mouse-Monoclonal Anti-LDHB, 1:100, sc-100775, Santa Cruz Biotechnology Inc; Mouse-Monoclonal Anti-MCT-1, 1:500, GT14612, Genetex; Mouse-Monoclonal Anti-MCT-4, 1:100, sc-376140, Santa Cruz Biotechnology Inc; Mouse-Monoclonal Anti-PDH-E1α, 1:100, sc-377092, Santa Cruz Biotechnology Inc; Mouse-Monoclonal Anti-citrate synthase, 1:100, sc-390693, Santa Cruz Biotechnology Inc; Mouse-Monoclonal Anti-IDH3A, 1:100, sc-398021, Santa Cruz Biotechnology Inc; DyLight 488 Goat anti-mouse IgG H&L, 1:1000, DI-1488, Vector Laboratories; DyLight 549 Goat anti-rabbit IgG H&L, 1:1000, DI-1549, Vector Laboratories; Alexa Fluor 488 Goat anti-rat IgG H&L, 1:1000, ab150157, Abcam. A laser scanning confocal microscope system (Zeiss LSM710; Carl Zeiss) was used to acquire images. The cross-section images of 63× magnification (135 × 135 μm) were used to measure quantitative Schwann cell counts and axonal fluorescence intensity. HK1, PFK1, MCT1, and MCT4/S-100B/DAPI co-positive cells were measured under four fields as HK-1/PFK-1/LDHA/LDHB/MCT-1/MCT-4 positive Schwann cells, and the total number of cells was counted. The axonal fluorescence intensity of HK-1, PFK-1, LDHA, LDHB, PDH-E1α, CS, and IDH3A were measured by using ImageJ, as previously described (Jia et al., 2021). Briefly, the average intensity within 100 axons surrounded by Schwann cells (S-100B) per mouse was measured with subtraction of the background intensity. Imaging analyses were performed in a blinded manner regarding the treatment status of the nerve. All others were observed in each of the three sections of at least three individuals.

### Mass spectrometry

Transection was performed on the right side, whereas the uninjured contralateral left side was used as a control. The entire distal sciatic nerve stump was analyzed at a continuous distance of 7 mm from the sectional end. The tissue samples were dissolved in ice-cold 80% methanol at a concentration of 100 mg/ml, sonicated five times (30 s of sonication and 30 s of cooling) using a BIORUPTOR (Cosmo Bio Co, Ltd), and centrifuged at 21,500 × *g* for 5 min at 4°C. The supernatant fluids were evaporated to dryness. After dissolving in 50 μl of 0.1% formic acid, the samples were subjected to liquid chromatography mass spectrometry analysis. Metabolites in the glycolytic system, TCA cycle, ATP, and NAD^+^ were analyzed employing liquid chromatography-mass spectrometry using LCMS-8040 instruments (Shimadzu). The sample was separated by reverse phase ion-pair chromatography using an ACQUITY UPLC BEH C18 column (100 × 2.1 mm, 1.7-μm particle size, Waters). The mobile phase consisted of solvent A (15 mm acetic acid and 10 mm tributylamine in 3% methanol) and solvent B (methanol), and the column oven temperature was 40°C. The gradient elution program was as follows: a flow rate of 0.3 ml/min: 0–3 min, 0% B; 3–5 min, 0–40% B; 5–7 min, 40–100% B; 7–10 min, 100% B; 10.1–14 min, 0% B. Parameters for the negative electrospray ionization source (ESI) mode under multiple reaction monitoring (MRM) were as follows; drying gas flow rate, 15 l/min; nebulizer gas flow rate, 3 l/min; DL temperature, 250°C; and heat block temperature, 400°C; collision energy (CE), 230 kPa. Data processing was conducted using the LabSolutions LC-MS software (Shimadzu).

### Local administration of inhibitor

Several inhibitors were locally administered to the sciatic nerve, as described in a previous report (Chung et al., 2019). A schematic illustration of the experimental design is shown in Extended Data Figure 7-1*a*. Briefly, under general anesthesia, the bilateral sciatic nerves of GO-ATeam2 mice were exposed and only the right sciatic nerve was cut (transection side). Immediately after axotomy, the distal nerve stump was covered with a gelatin sponge (873322, LTL Pharma) soaked with inhibitors and a 3-mm PVC tube to prevent leakage of the solution, whereas the left intact sciatic nerve was only covered with a gelatin sponge soaked with inhibitors (sham side). The fascia and skin were then sutured tightly. One day (24 h) after the surgical procedure, ATP imaging with two-photon microscopy or harvesting of sciatic nerve lesions was performed. All procedures were performed under a stereomicroscope (L-0950D; Inami Corporation). We used 2-Deoxy-D-glucose (2-DG) as the indirect hexokinase (HK) inhibitor, MSDC-0160 as the mitochondrial pyruvate carrier (MPC) inhibitor, and a-cyano-4-hydroxycinnamic acid (4-CIN) as a comprehensive inhibitor of MCTs (Extended Data Fig. 7-1*b*). Before proceeding to the main experiment, we preliminarily measured the change of ATP levels in the distal nerve stump for 120 min intermittently, by using two concentrations of all inhibitors (Extended Data Fig. 7-1*c*,*d*). The concentrations of inhibitors were determined from previous studies (Press and Milbrandt, 2008; Miranda-Goncalves et al., 2013; Tsuyama et al., 2013; Patterson et al., 2014; Anamitra Ghosh, 2016; Ichihara et al., 2017; Babetto et al., 2020). Then, we used higher concentrations for all inhibitors in the main experiments. The concentrations of the inhibitors were 150 mm 2DG (D8375, Sigma-Aldrich), 100 μm MSDC-0160(HY-100550, Med Chem Express), and 10 mm 4-CIN (70990, Sigma-Aldrich). For all inhibitors, the solvent was dimethyl sulfoxide (DMSO) diluted to a 0.1% concentration with saline, and only the DMSO group was used as the solvent.

### Local administration of EP

Ethyl pyruvate (EP; 051-05292, Wako) was used because of the poor stability of pyruvate, and the concentrations were applied at 3, 10, 30, and 100 mm based on previous reports (Park et al., 2015; Chung et al., 2019). The method of topical administration was the same as that of the inhibitor. Briefly, under general anesthesia, the right sciatic nerve was exposed and performed axotomy. Immediately after the axotomy, the distal nerve stump was covered with a gelatin sponge soaked with EP, diluted with saline as the solvent. Only saline was defined as the saline group. Two days (48 h) after the surgical procedure, ATP imaging with two-photon microscopy or harvesting of sciatic nerve lesions was performed.

### Statistical analysis

Statistical analysis was performed using EZR (version 1.40, Saitama Medical Center, Jichi Medical University, Saitama, Japan), a graphical user interface for R (version 4.0.4, The R Foundation for Statistical Computing). Sample sizes were selected based on those generally used in this research field. Data sets with a normal distribution and equal variances are presented as mean ± SD, whereas the others are represented as mean and interquartile ranges. A two-tailed *t* test, Spearman’s ρ correlation coefficient and spearman test, or Mann–Whitney *U* test was used to compare two groups according to the distribution and variance of the data. One-way ANOVA followed by Tukey’s *post hoc* test was used to compare more than two groups. Two-way ANOVA followed by Tukey’s *post hoc* test was used to compare the data, which were affected from two different factors. In all statistical analyses, *p* values <0.05 were considered significant.

### Data accessibility statement

The data that support the findings of this study are available from the corresponding author on reasonable request.

## Results

### Spatiotemporal decrease of ATP levels in the distal nerve stump and mitochondrial degeneration in axon

To clarify the changes in intracellular ATP in WD, we first examined ATP levels in the *GO-ATeam2* knock-in rat sciatic nerve transection model. In the six ROIs, we measured whole-nerve ATP levels intermittently until 360 min after axotomy in the same individuals (Fig. 1*a*,*b*). Distal nerve stump ATP levels (ROI ^#^5 and ^#^6) did not decrease for 360 min (Fig. 1*a*,*b*). Only the sectional end (ROIs ^#^3 and ^#^4) revealed a significant decrease in ATP levels 5 min after axotomy (Fig. 1*a*,*b*).

**Figure 2.**
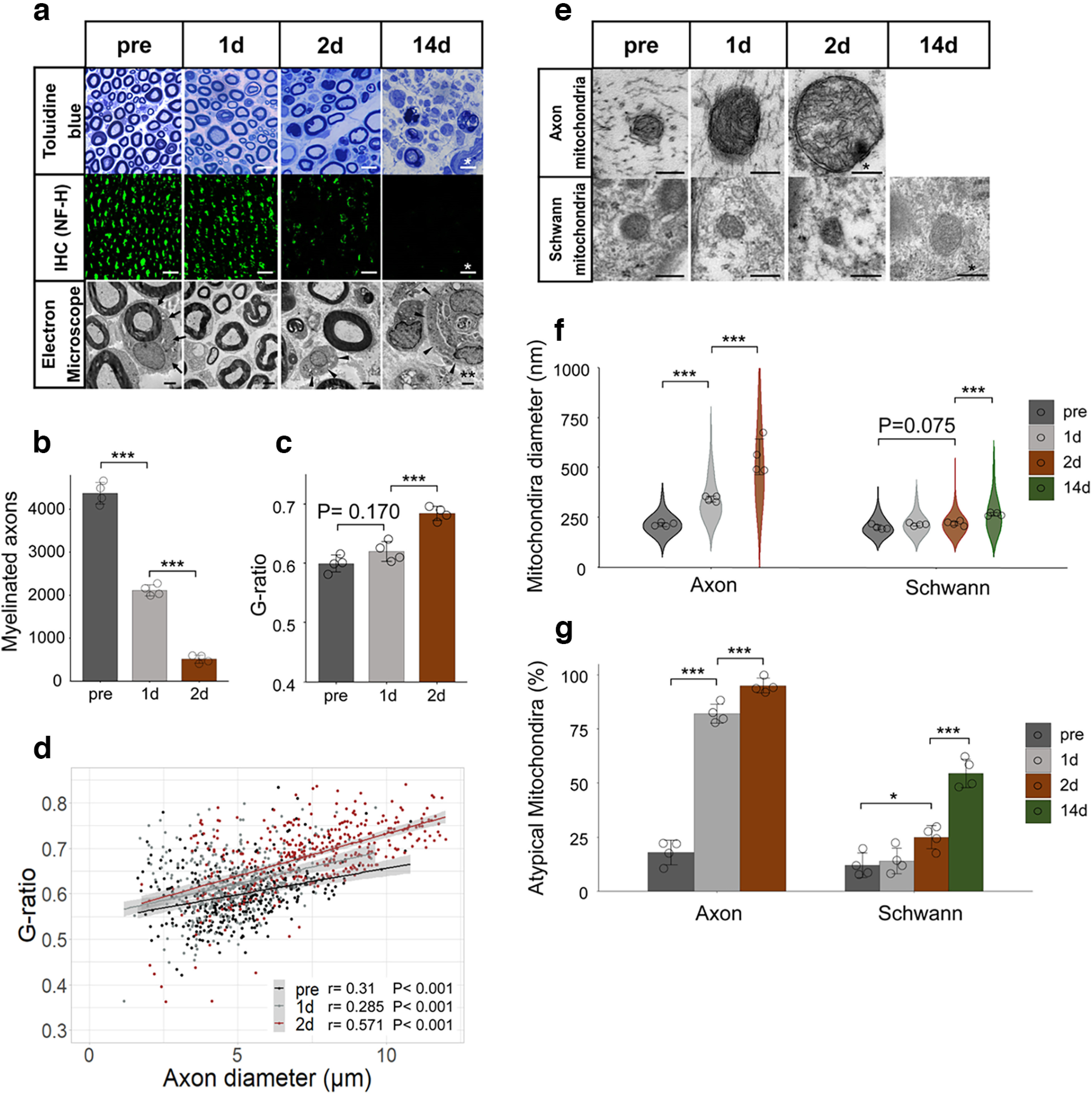
Wallerian degeneration and mitochondrial degeneration in axon for rat’s distal nerve stump. ***a***, Representative images of toluidine blue staining, immunohistochemistry (NF-H), electron microscopy, before (pre) and 1, 2, and 14 d after axotomy. Immunohistochemistry was obtained the same results for three individuals. Scale bar, * 10 μm, ** 2 μm. Arrows, myelinated axon; arrowheads, demyelinated Schwann cell. ***b***, Quantification of the myelinated axon. ***c***, ***d***, Quantification of the G-ratio [***c***: cumulative G-ratio per individual, ***d***: scatter plot, Spearman’s rank correlation coefficient and *p* values showing G-ratio of individual myelinated axons against axon diameter (*n* = 400)]. The histologic WD was observed significantly from 2 d after axotomy. In axons, before (pre) and 1 and 2 d after axotomy were evaluated, except for after 14 d with completely collapsed axon morphology (*n* = 4 rat per group). ***e***, Representative images of mitochondrial findings, before (pre) and 1, 2, and 14 d after axotomy. Evaluation was performed in axons and Schwann cells separately. Scale bar, * 200 nm. ***f***, Violin plot of mitochondrial diameter (nm; *n* = 200). Cumulative mitochondria diameter per individual are also shown (*n* = 4 rat per group). ***g***, Quantitative analysis of atypical mitochondria (%; *n* = 4 rat per group). In axons, mitochondria were significantly degenerated from 1 d after axotomy, whereas in Schwann cells, mitochondria were not significantly degenerated until 14 d after axotomy. All histologic evaluations were performed 3 mm distal to the sectional end and corresponding uninjured nerve. Error bars indicate SD; *p < 0.05, ***p < 0.001, one-way ANOVA followed by the Tukey’s *post hoc* test (for comparison of axons and Schwann cells, respectively).

Next, we measured ATP levels before (pre), 1 d (24 h), 2 d (48 h), and 14 d after axotomy in separate individuals (Fig. 1*c*,*d*). Two days after axotomy, distal nerve stump ATP levels (ROI ^#^5) showed a significant decrease (Fig. 1*c*,*d*). The most distal (ROI ^#^6) ATP levels showed a decreasing tendency, but not significant (Fig. 1*c*,*d*). We also evaluated the histology 3 mm distal from the sectional end (Extended Data Fig. 1-1, corresponding to ROI ^#^6). Two days after axotomy, the number of myelinated axons significantly decreased (Fig. 2*a*,*b*), and the G-ratio significantly increased (Fig. 2*a*,*c*,*d*), indicating WD progression. These findings suggest that the decrease in ATP levels is associated with histologic progression of WD in the distal nerve stump.

To examine the primary ATP source in the distal nerve stump, we evaluated mitochondrial diameter and atypicality. The main component units of the sciatic nerve were evaluated separately in the axons and Schwann cells. In axons, mitochondria showed enlarged diameters and progressed atypicality from 1 d after axotomy, whereas in Schwann cells, they showed critical and significant change between 2 and 14 d (Fig. 2*e–g*). These findings indicated that the distal nerve stump caused the mitochondrial degeneration in axons.

### Gradual decrease of ATP levels in the distal nerve stump and axonal degeneration

**Figure 3.**
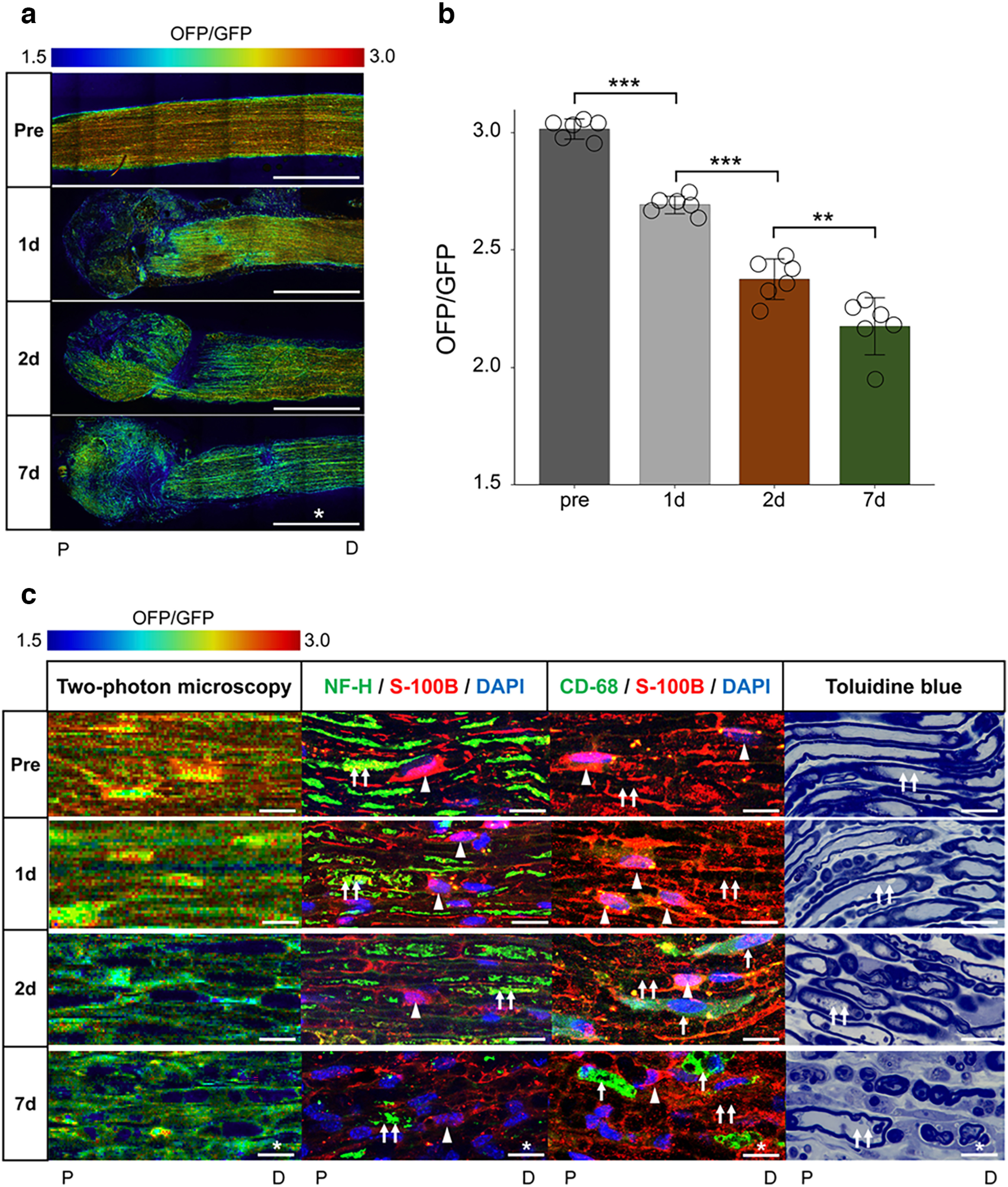
The gradual decrease of mice longitudinal nerve cross section ATP levels in the distal nerve stump, corresponds to histology. ***a***, Representative ATP images in the longitudinal section before (pre) and 1, 2, and 7 d after axotomy. Scale bar, * 1 mm. P, proximal. D, distal. ***b***, The change of ATP levels with measured ROIs (*n* = 6 mice per group). Distal nerve stump ATP levels were significantly decreased from 1 d after axotomy. Error bars indicate SD; **p < 0.01, ***p < 0.001, one-way ANOVA followed by the Tukey’s *post hoc* test. ***c***, Representative enlarged images of two-photon microscopy, immunohistochemistry (left, NF-H/S-100B/DAPI; right, CD68/S-100B/DAPI), and toluidine blue staining, before (pre) and 1, 2, and 7 d after axotomy. Scale bar, * 10 μm. Arrows, macrophage; arrowheads, Schwann cell; double arrows, axonal soma. The ROI for measuring ATP levels was set at 3 mm distal from the sectional end, the center of the longitudinal cross-section, with a 300 × 300-μm square (Extended Data Fig. 3-1). The OFP/GFP ratios ranged from 1.5 to 3.0. All histologic evaluations were performed with the same results for three individuals, 3 mm distal from the sectional end, and the corresponding uninjured nerve. ROI, region of interest.

Next, to assess ATP levels of the sciatic nerve components in the distal nerve stump, we performed the two-photon microscopic analysis in the center of the longitudinal section before (pre), 1 d (24 h), 2 d (48 h), and 7 d after axotomy of *GO-ATeam2* knock-in mice. Two-photon microscopy allow us to remove interference with ATP levels by epineurium tissue. We measured intracellular ATP levels 3 mm distal from the sectional end with a 300 × 300-μm square ROI. The distal nerve stump ATP levels showed a gradual and significant decrease (Fig. 3*a*,*b*). The immunohistochemistry for neurofilament and toluidine blue staining presented the low detection and fragmentation, indicating the axonal degeneration over time 2 d after axotomy (Fig. 3*c*, double arrows). However, S-100B-positive Schwann cell bodies were detected up to 7 d after axotomy (Fig. 3*c*, arrowhead). We also detected infiltrated CD68 macrophages 2 d after axotomy (Fig. 3*c*, arrow). Although the sciatic nerve components included in axons, Schwan cells, and macrophages 2 d after axotomy, we could not detect the specific components with ATP levels same as preaxotomy (Fig. 3*c*). These findings suggested that a gradual decrease in ATP levels in the distal nerve stump was preceded by WD.

10.1523/ENEURO.0353-22.2023.f3-1 Extended Data Figure 3-1 Experimental system for ATP levels with two-photon microscopy and histological analysis in mice. A schematic illustration of center of the longitudinal nerve cross-section ATP levels measurement for GO-ATeam2 mouse. All of fluorescence emission in the GO-ATeam2 probe was captured with two-photon microscopy. ATP levels were measured in each ROIs (*** 300-μm square), 3 mm distal from the sectional end (broken line). OFP/GFP ratio ranges were from 1.5 to 3.0. The histological analysis was performed 3 mm distal from the sectional end. Scale bar, * 1 mm;*** 500-μm square ROI. P, proximal. D, distal. Download Figure 3-1, TIF file.

### Activation of the glycolytic system after axotomy

**Figure 4.**
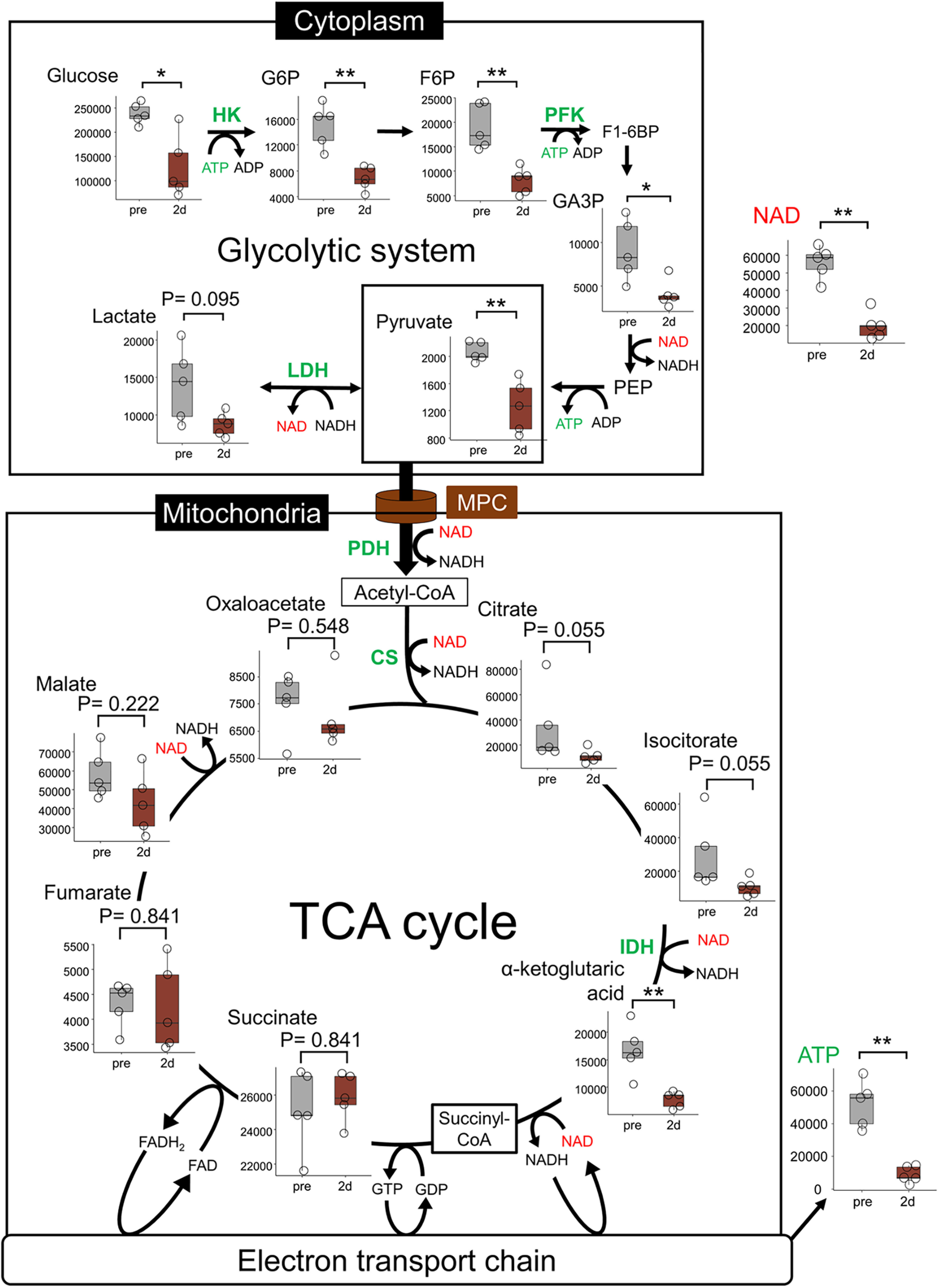
Activation of the glycolytic system. Diagram of energy metabolism pathways and mass spectrometry before and 2 d (48 h) after axotomy. MPC transports pyruvate, the end-product of the glycolytic system, for metabolism in the TCA cycle. Mass spectrometry was performed for the metabolites of glycolysis and the TCA cycle, the end-product ATP, and the coenzyme NAD^+^. Glycolytic intermediates, NAD^+^ and ATP were significantly decreased after axotomy. These results are shown in a boxplot containing the interquartile range and centered bars as the median (*n* = 5 mice per group). All examinations were performed continuously 7 mm from the sectional end of the right sciatic nerve with transection and the corresponding lesion on the left side. *p < 0.05, **p < 0.01, Mann–Whitney *U* test. CS, citrate synthase; F6P, fructose 6-phosphate; G6P, glucose-6-phosphate; GA3P, glyceraldehyde-3-phosphate; HK, hexokinase; IDH, isocitrate dehydrogenase; LDH, lactate dehydrogenase; NAD^+^, nicotinamide adenine dinucleotide; PFK, phosphofructokinase; PDH, pyruvate dehydrogenase; TCA cycle, tricarboxylic acid cycle.

The main metabolic pathways of ATP production include the glycolytic system in the cytoplasm and the TCA cycle in the mitochondria. To clarify the ATP production pathway in the distal nerve stump, we performed mass spectrometry for energy metabolites before (pre) and 2 d (48 h) after axotomy. The metabolites of the glycolytic system, glucose, glucose-6-phosphate (G6P), fructose 6-phosphate (F6P), glyceraldehyde-3-phosphate (GA3P), and pyruvate, showed a significant decrease after axotomy (Fig. 4, upper square). However, lactate, which is reversible metabolite from pyruvate, showed a nonsignificant decrease. These results suggested that the distal stump activated the glycolytic pathway.

Under aerobic conditions, the MPC transports pyruvate from the cytoplasm to the mitochondria for metabolism in the TCA cycle (Bricker et al., 2012; Halestrap, 2012). Metabolites of the first part of the TCA cycle in the mitochondria, citrate and isocitrate showed nonsignificant decrease while α-ketoglutaric acid showed significant decrease after axotomy (Fig. 4, lower square). ATP and NAD^+^ levels showed a significant decrease after axotomy (Fig. 4). Mass spectrometry also showed a decrease in ATP levels in the distal nerve stump, consistent with the results of our *in vivo* imaging.

### Schwann cells activate the glycolytic system and monocarboxylate transport after axotomy

The mass spectrometry could not evaluate the respective metabolic changes of Schwann cells and axons as it reflected the whole intracellular changes of the distal nerve stump. Therefore, we next performed immunohistochemical evaluation with cell type-specific antibodies and metabolic enzymes.

**Figure 5.**
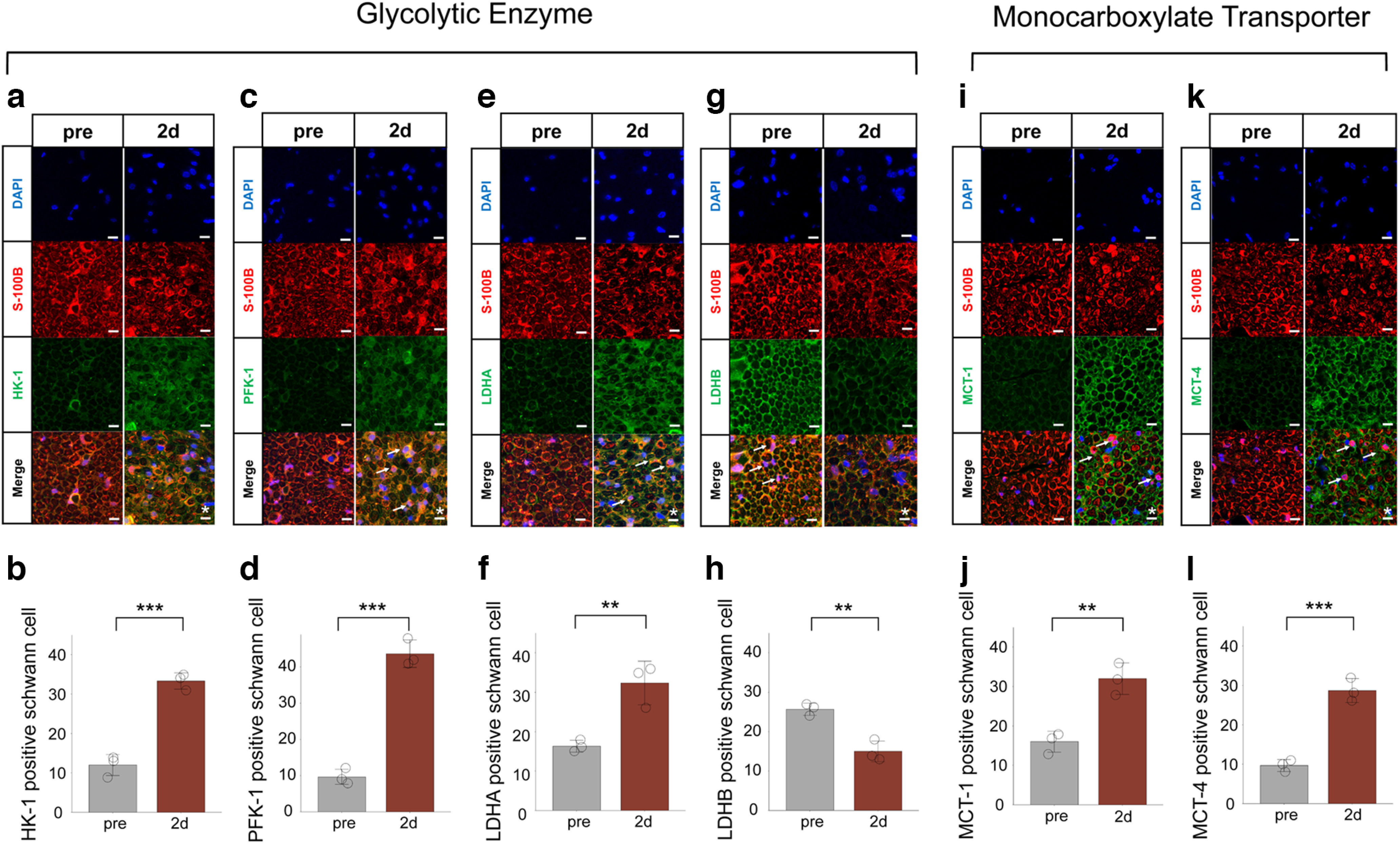
Activation of the glycolytic system and MCTs in Schwann cell after axotomy. ***a***, ***c***, ***e***, ***g***, Representative cross-section images of immunohistochemistry for glycolytic enzyme (***a***: HK-1, ***c***: PFK-1, ***e***: LDHA, ***g***: LDHB) before and 2 d after axotomy. ***b***, ***d***, ***f***, ***h***, The number of HK-1 (***b***), PFK-1 (***d***), LDHA (***f***), and LDHB (***h***) positive Schwann cells (*n* = 3 mice per group). HK-1, PFK1, and LDHA positive Schwann cells were significantly increased after axotomy, whereas LDHB positive Schwann cells were significantly decreased. ***i***, ***k***, Representative images of immunohistochemistry for MCT (***e***: MCT-1, ***g***: MCT-4) before and after axotomy. ***j***, ***l***, The number of MCT-1 (***j***) or MCT-4 (***l***) positive Schwann cells (*n* = 3 mice per group). MCT-1 and MCT-4 positive Schwann cells were significantly increased after axotomy. All histologic evaluations were performed 3 mm distal to the sectional end and corresponding uninjured nerve. Error bars indicate SD. Arrows, HK-1/PFK-1/LDHA/LDHB/MCT-1/MCT-4 positive Schwann cells; scale bar, 10 μm. *p < 0.05, **p < 0.01, two-tailed *t* test. HK, hexokinase; LDHA, lactate dehydrogenase A subunit lactate; LDHB, lactate dehydrogenase B subunit lactate; dehydrogenase; MCT-1, monocarboxylate transporters 1; MCT-4, monocarboxylate transporters 4; PFK, phosphofructokinase.

In Schwann cells, we evaluated the activity of glycolytic system and MCTs after axotomy. We performed immunohistochemical analysis for glycolytic enzymes (HK-1, hexokinase 1; PFK-1, phosphofructokinase 1; LDHA, lactate dehydrogenase A subunit; LDHB, lactate dehydrogenase B subunit) and MCTs (1/4) before (pre) and 2 d (48 h) after axotomy, and measured the number of glycolytic enzymes and MCT-positive Schwann cells. HK-1, PFK1, and LDHA positive Schwann cells showed a significant increase after axotomy (Fig. 5*a–f*). MCT-1 and MCT-4 positive Schwann cell numbers also showed a significant increase (Fig. 5*i–l*). However, LDHB positive Schwann cells showed a significant decrease after axotomy (Fig. 5*g*,*h*). LDHA is the enzyme that converts pyruvate to lactate and NAD^+^, whereas LDHB is the enzyme that reacts in the opposite direction (Doherty and Cleveland, 2013), and NAD^+^ is used for working glycolysis (Fantin et al., 2006; Diaz-Garcia et al., 2017; Urbanska and Orzechowski, 2019; Osis et al., 2021). These results suggest that the glycolytic system in Schwann cells and MCT-induced monocarboxylate transport to axons were activated in the distal nerve stump.

### Axons activate the glycolytic system and inactivate the TCA cycle after axotomy

**Figure 6.**
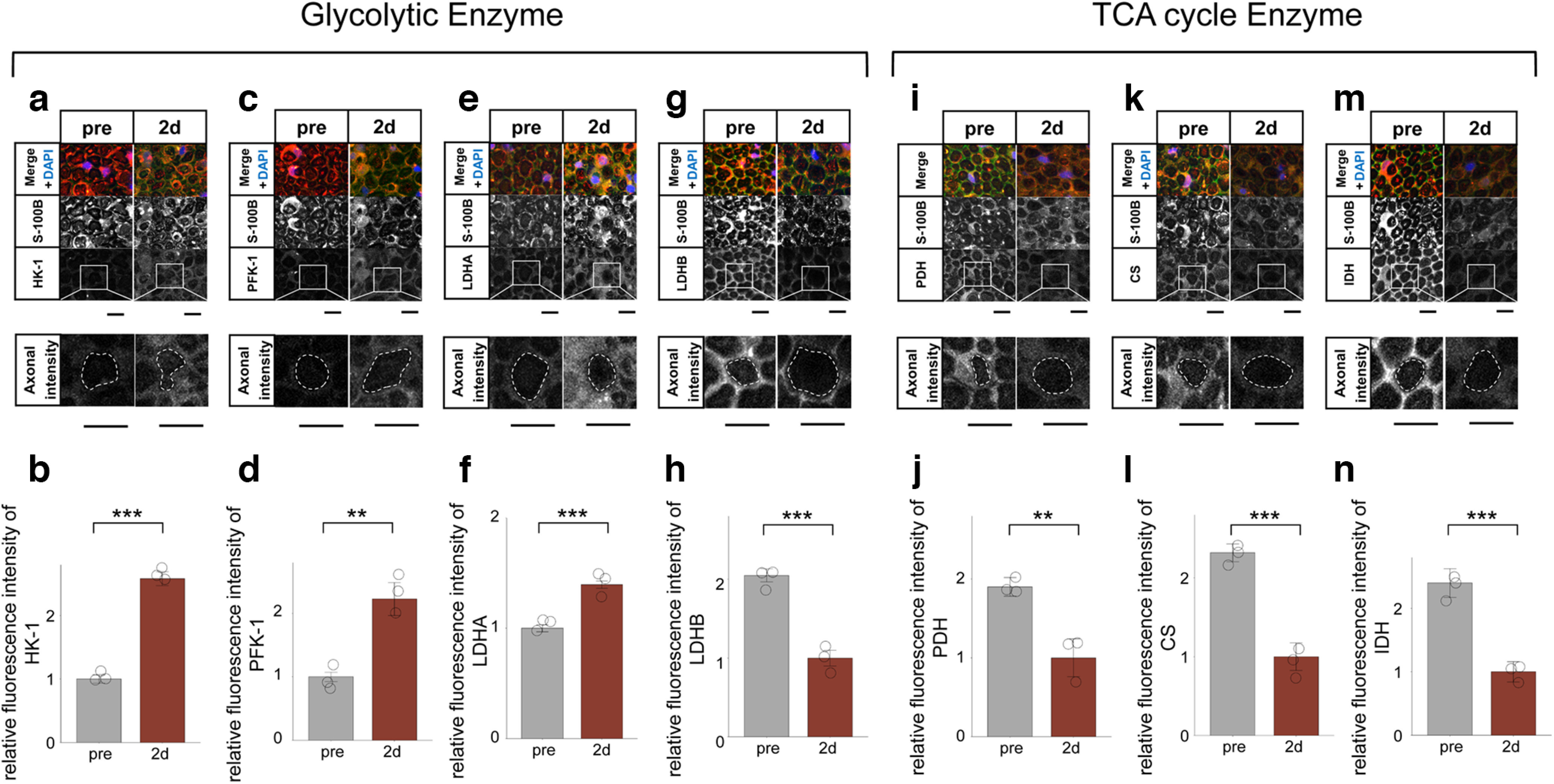
Activation of the glycolytic system and inactivation of the TCA cycle in axons after axotomy. ***a***, ***c***, ***e***, ***g***, Representative cross-section images of immunohistochemistry for glycolytic enzyme (***a***: HK-1, ***c***: PFK-1, ***e***: LDHA, ***g***: LDHB) before and 2 d after axotomy. The axonal fluorescence intensity in the dashed line was measured. ***b***, ***d***, ***f***, ***h***, The axonal fluorescence intensity of HK-1 (***b***), PFK-1 (***d***), LDHA (***f***), and LDHB (***h***; *n* = 3 mice per group). HK-1, PFK1, and LDHA fluorescence intensity was significantly increased after axotomy, whereas LDHB was significantly decreased. ***i***, ***k***, ***m***, Representative images of immunohistochemistry for TCA cycle enzyme (***i***: PDH-E1α, ***k***: CS, ***m***: IDH3A) before and after axotomy. The axonal fluorescence intensity in the dashed line was measured. ***j***, ***l***, ***n***, The axonal fluorescence intensity of PDH-E1α (***j***), CS (***k***), and IDH3A (***n***; *n* = 3 mice per group). PDH-E1α, CS, and IDH3A fluorescence intensity was significantly decreased after axotomy. All histologic evaluations were performed 3 mm distal to the sectional end and corresponding uninjured nerve. Error bars indicate SD scale bar, 10 μm. *p < 0.05, **p < 0.01, ***p < 0.001, two-tailed *t* test. CS, citrate synthase; IDH, isocitrate dehydrogenase; HK, hexokinase; LDHA, lactate dehydrogenase A subunit lactate; LDHB, lactate dehydrogenase B subunit lactate; dehydrogenase; PFK, phosphofructokinase; PDH, pyruvate dehydrogenase; TCA cycle, tricarboxylic acid cycle.

In axons, we evaluated the activity of glycolytic system and TCA cycle after axotomy. We performed immunohistochemical analysis for glycolytic enzymes (HK-1, PFK-1, LDHA, LDHB), and TCA cycle enzymes (PDH, pyruvate dehydrogenase; CS, citrate synthase; IDH, isocitrate dehydrogenase) before (pre) and 2 d (48 h) after axotomy, and measured the fluorescence intensity within axons surrounding Schwann cell-specific staining (S-100B). Interestingly, the relative intensity of HK-1, PFK1, and LDHA showed a significant increase after axotomy (Fig. 6*a–f*). Conversely, the relative intensity of LDHB, PDH-E1α, CS, and IDH3A showed a significant decrease after axotomy (Fig. 6*g–n*). In the distal nerve stump, we showed that the glycolytic system was activated not only in Schwann cells but also in axons. Furthermore, TCA cycle was inactivated in axons. These results suggest that even in axons, the metabolic source of ATP is mainly on glycolytic system in the distal nerve stump. This theory is also supported from mitochondrial degeneration in axons (Fig. 2*e–g*).

### Inhibition of the glycolytic system and MCT-induced monocarboxylate transport decrease ATP levels and progress WD after axotomy, whereas TCA cycle does not

In the distal nerve stump, we showed that Schwann cells activate the glycolytic system and MCT-induced monocarboxylate transport, axons inactivate TCA cycle while activating the glycolytic system, resulting a gradual decrease in ATP levels. Then, we hypothesized that the inhibition of the glycolytic system and MCT-induced monocarboxylate transport progress WD, whereas the inhibition of the TCA cycle did not. To prove this hypothesis, we performed *in vivo* inhibitor experiments. We tried several energy metabolic inhibitors to axotomized right sciatic nerve (transection side) and served left (sham side), and evaluated progressive WD after 1 d (24 h).

**Figure 7.**
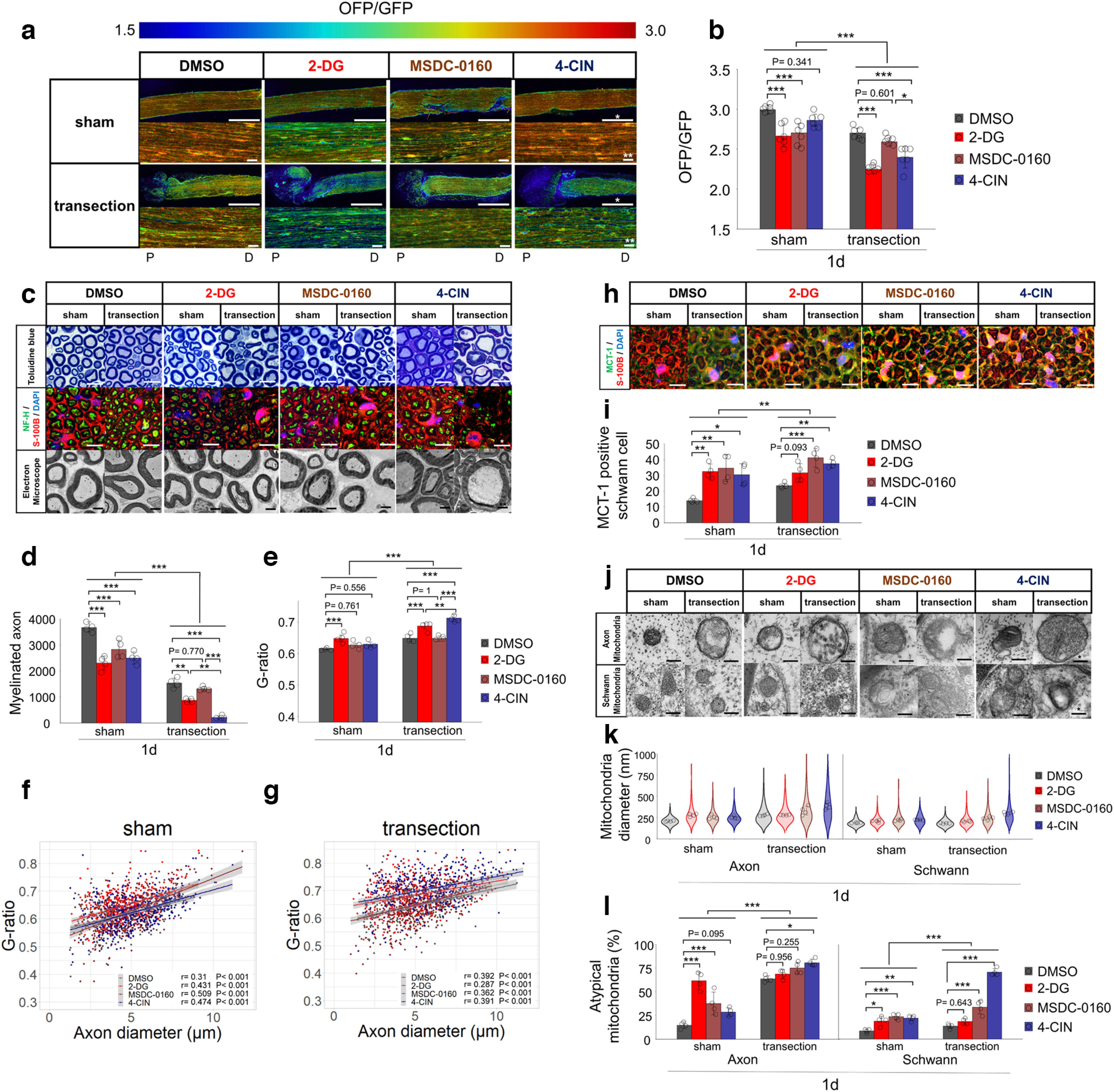
Inhibition of the glycolytic system or MCTs progress Wallerian degeneration with decreased ATP levels, but MPC does not. ***a***, Representative ATP images for each inhibitor in both sham and transection side. For each experiment, lower figures are enlarged image with measured ROI. Upper figures present overall distal nerve stump ATP image, from the sectional end. Scale bar, * 1 mm, ** 10 μm. P, proximal. D, distal. ***b***, The distal nerve stump ATP levels with measured ROI (*n* = 6 mice per group). ***c***, Representative images of toluidine blue staining, immunohistochemistry (NF-H/S-100B/DAPI), electron microscopy for each inhibitor in both sham and transection side. Scale bar, * 10 μm, ** 2 μm. ***d***, Quantification of the myelinated axon. ***e–g***, Quantification of the G-ratio [***e***: cumulative G-ratio per individual; ***f***, ***g***: scatter plot, Spearman’s rank correlation coefficient and *p* values showing G-ratio of individual myelinated axons against axon diameter (*n* = 400) in both (***f***) sham and (***g***) transection side] (*n* = 4 mice per group). ***h***, Representative images of immunohistochemistry (MCT-1/S-100B/DAPI) for each inhibitor in both sham and transection side. Scale bar, * 10 μm. ***i***, The number of MCT-1 positive Schwann cells (*n* = 4 mice per group). ***j***, Representative images of mitochondrial findings for each inhibitor in both sham and transection side. Evaluation was performed in axons and Schwann cells separately. Scale bar, * 200 nm. ***k***, Violin plot of mitochondrial diameter (nm; *n* = 200). Cumulative mitochondria diameter per individual are also shown (*n* = 4 rat per group). ***l***, Quantitative analysis of atypical mitochondria (%; *n* = 4 mice per group). All examinations were performed 1 d (24 h) after inhibitor administration and evaluated 3 mm distal from the sectional end and the corresponding uninjured nerve (Extended Data Fig. 7-1*a*). The concentrations of all inhibitors were determined from preliminarily experiments (Extended Data Fig. 7-1*b–d*), by measuring ATP levels 3 mm distal to the sectional end, with a 500 × 500-μm square (Extended Data Fig. 7-2). The ROI for measuring ATP levels was set at 3 mm distal from the sectional end, the center of the longitudinal nerve cross-section, with a 300 × 300-μm square (Extended Data Fig. 3-1). The OFP/GFP ratios ranged from 1.5 to 3.0. Immunohistochemistry showed the same results for the four individuals. A summary of the inhibition experiments is provided in Extended Data Table 7-1. Error bars indicate SD; *p < 0.05, **p < 0.01, ***p < 0.001, two-way ANOVA followed by the Tukey’s *post hoc* test (for comparison of sham group and transection group, respectively). No statistical data were available for mitochondrial diameter because there was no equal distribution. 2-DG, 2-deoxyglucose; 4-CIN, a-cyano-4-hydroxycinnamic acid; MCT-1, monocarboxylate transporters 1; MSDC-0160, mitochondrial pyruvate carrier (MPC) inhibitors; ROI, region of interest.

First, we performed a 2-DG treatment to inhibit the glycolytic system. The 2-DG group showed a further decrease in ATP levels on both the sham and transection sides compared with the DMSO group (Fig. 7*a*,*b*). The 2-DG group also showed a decrease in the number of myelinated axons and an increase in the G-ratio on both sides (Fig. 7*c–g*). On both sides, the 2-DG group also showed an increase in MCT1-positive Schwann cells (Fig. 7*h*,*i*). In addition, only on the sham side of the 2-DG group show a progression of mitochondrial atypicality (Fig. 7*j*,*k*). Interestingly, the transection side of the 2-DG group did not show further progression of mitochondrial atypicality in Schwann cells and axons (Fig. 7*j*,*k*). In the distal nerve stump, inhibition of the glycolytic system decreased ATP levels and progressed WD but had nominal effect on mitochondrial degeneration in axons.

10.1523/ENEURO.0353-22.2023.f7-1 Extended Data Figure 7-1 Inhibition experiments following the test of two concentrations and measuring ATP levels until 120 min after axotomy. ***a***, A schematic illustration of inhibition experiments. The following inhibitors was used; 2-DG, MSDC-1060, and 4-CIN. The control was set as 0.1% DMSO dissolved in saline. Bilateral sciatic nerves were exposed, and transection side as right, sham side as left. Immediately after the transection of right sciatic nerve, bilateral sciatic nerves were covered with a gelatin sponge soaked with inhibitors. One day after the surgical procedure, ATP levels and histology were evaluated in 3 mm distal from the sectional end. ***b***, Energy metabolic interactions between Schwann cells and axon, and the working point of the inhibitors. Monocarboxylate produced by the Schwann cell glycolytic system is transported to axons through MCTs. Pyruvate, the end product of the glycolytic system, is transported to the mitochondrial matrix through MPC and metabolized in the TCA cycle and electron transport chain. 2-DG is a glycolysis inhibitor, MSDC-0160 is the MPC inhibitor, and 4-CIN is the comprehensive MCTs inhibitor. ***c***, Representative intracellular distal nerve stump ATP levels for each inhibitor compared to the DMSO group, before (pre) and 15, 30, 45, 60, 75, 90, 105, and 120 min after axotomy. Scale bar, * 1mm. P, proximal. D, distal. ***d***, ATP reduction response for two different concentrations of each inhibitor, compared to the DMSO group. Two concentrations of several inhibitors were preliminary tested. *p < 0.05, two-way ANOVA followed by the Tukey’s *post hoc* test. The ROI for measuring ATP levels was set at 3 mm distal to the sectional end, with a 500 × 500-μm square (Extended Data Fig. 7-2). A higher concentration (150 mm 2DG, 100 μm MSDC-0160, and 10 mm 4-CIN) was applied for experiment in all inhibitors. The OFP/GFP ratios ranged from 1.5 to 3.0. Error bars indicate SD. *p < 0.05, one-way ANOVA followed by Tukey’s *post hoc* test. Download Figure 7-1, TIF file.

10.1523/ENEURO.0353-22.2023.f7-2 Extended Data Figure 7-2 Experimental system for ATP levels with fluorescence microscopy in mice. A schematic illustration of whole nerve ATP levels measurement for GO-ATeam2 mouse. All of fluorescence emission in the GO-ATeam2 probe was captured with fluorescence microscopy. ATP levels were measured in each ROIs (** 500-μm square), 3 mm distal from the sectional end (broken line). OFP/GFP ratio ranges were from 1.5 to 3.0. The histological analysis was performed 3 mm distal from the sectional end. Scale bar, * 1 mm;** 500-μm square ROI. *** 500-μm square ROI. P, proximal. D, distal. Download Figure 7-2, TIF file.

10.1523/ENEURO.0353-22.2023.tab7-1 Extended Data Table 7-1 The summary of the inhibition experiments. The downward arrow means a decrease in the value of each variable relative to the horizontal arrow, whereas upward arrow means an increase. The more numbers of arrows, the greater the change in value. 2-DG, 2-deoxyglucose; 4-CIN, a-cyano-4-hydroxycinnamic acid; MCT-1, monocarboxylate transporters 1; MSDC-0160, mitochondrial pyruvate carrier (MPC) inhibitors. Download Table 7-1, DOCX file.

Next, we performed MSDC-0160 treatment to inhibit MPC. On the sham side, the MSDC-0160 group showed a decrease in ATP levels (Fig. 7*a*,*b*) and a decrease in the number of myelinated axons, compared with the DMSO group (Fig. 7*c–g*). However, on the transection side, MSDC-0160 showed neither decrease in ATP levels (Fig. 7*a*,*b*) nor decrease number of myelinated axons and increase in G-ratio (Fig. 7*c–g*). On both sides, the MSDC-0160 group also showed an increase in MCT1-positive Schwann cells (Fig. 7*h*,*i*). On the sham side, the MSDC-0160 group showed progression of mitochondrial atypicality in Schwann cells, and axons (Fig. 7*i*,*j*). On the transection side, the MSDC-0160 group showed a further progression of mitochondrial atypicality in Schwann cells (Fig. 7*i*,*j*). These results revealed that the MPC inhibitor causes ATP depletion and WD progression only in uninjured nerves, but not in the distal nerve stump.

Finally, we performed 4-CIN treatment to inhibit MCT. On the transection side, the 4-CIN group showed a further decrease in ATP levels (Fig. 7*a*,*b*), a decrease in the number of myelinated axons, and an increase in the G-ratio compared with the DMSO or MSDC-0160 group (Fig. 7*c–g*). In particular, myelinated axons and the G-ratio showed the drastic progression of WD among all inhibitors (Fig. 7*c–g*). On both sides, the 4-CIN group also showed an increase in MCT1-positive Schwann cells (Fig. 7*h*,*i*), progression of mitochondrial atypicality in Schwann cells (Fig. 7*i*,*j*). The 4-CIN group showed further progression of mitochondrial atypicality in axons only on the transection side (Fig. 7*i*,*j*). These results indicate that the MCT inhibitor most strongly progressed WD after axotomy and decreased ATP levels. However, the MCT inhibitor did not induce WD and decreased ATP levels before axotomy, suggesting that MCT-induced monocarboxylate transport is important after axotomy to supply energy metabolism.

In summary, on the transection side, inhibition of the glycolytic system and MCT progressed WD, whereas inhibition of MPC did not progress WD. On the sham side, inhibition of the glycolytic system and MPC progressed WD, whereas inhibition of MCT showed less WD progression. These findings supported that the metabolic source of ATP in the distal nerve stump was mainly depended on glycolytic system via MCT-induced monocarboxylate transport. A summary of the inhibition experiments is provided in Extended Data Table 7-1.

### EP supplementation activates MCT-induced transport resulting in delay WD

**Figure 8.**
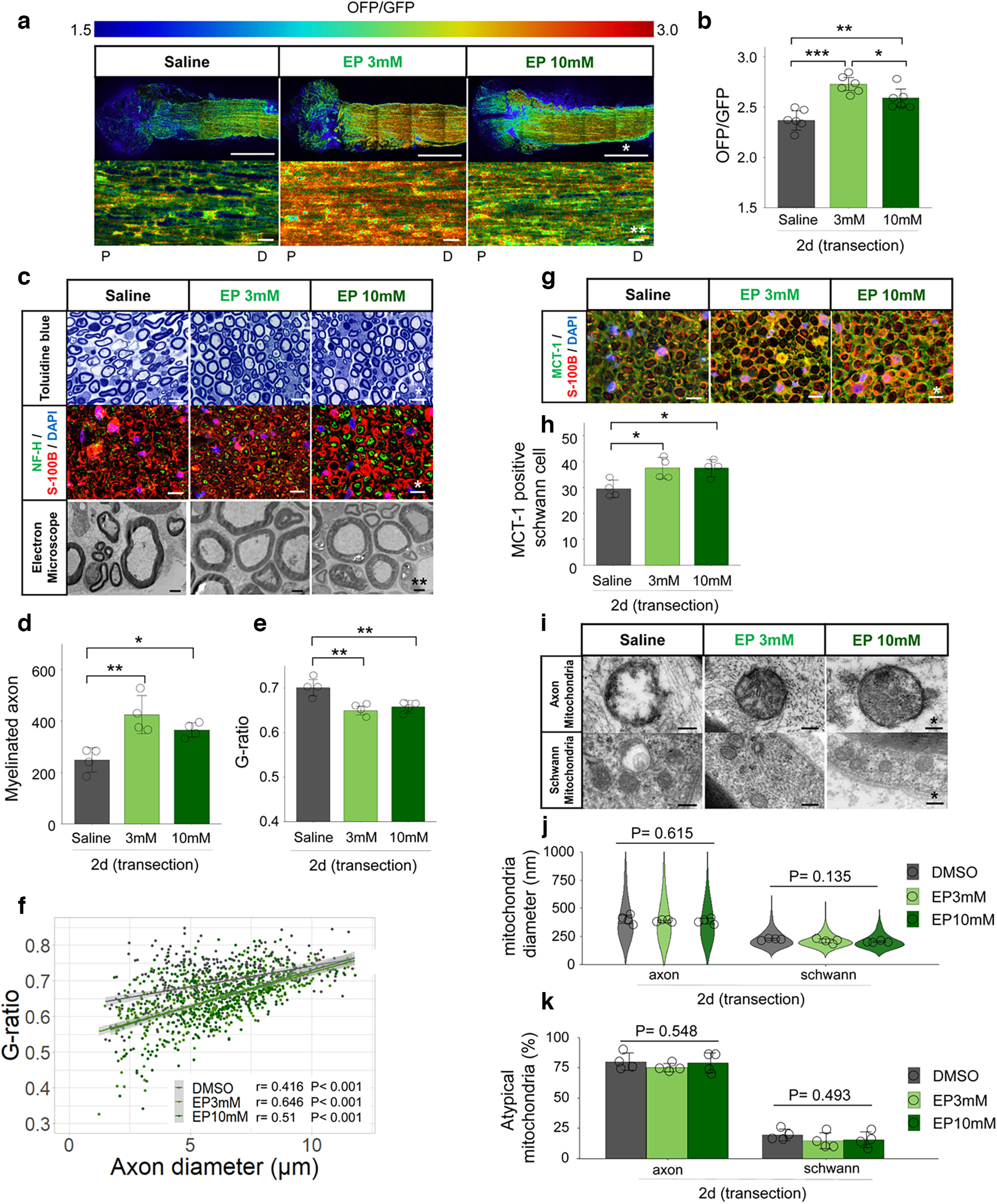
Ethyl pyruvate (EP) delays Wallerian degeneration with increased ATP levels. ***a***, Representative ATP images for each treatment. Lower figures are enlarged image in the measured ROI. Upper figures present overall distal nerve stump image, from the sectional end. Scale bar, * 1 mm, ** 10 μm. P, proximal. D, distal. ***b***, The distal nerve stump ATP levels with measured ROIs (*n* = 6 mice per group). ***c***, Representative images of toluidine blue staining, immunohistochemistry (NF-H/S-100B/DAPI), electron microscopy, for each treatment. Scale bar, * 10 μm, ** 2 μm. ***d***, Quantification of the myelinated axon. ***e***, ***f***, Quantification of the G-ratio [***e***: cumulative G-ratio per individual, ***f***: scatter plot, Spearman’s rank correlation coefficient and *p* values showing G-ratio of individual myelinated axons against axon diameter (*n* = 400)] (*n* = 4 mice per group). ***g***, Representative images of immunohistochemistry (MCT-1/S-100B/DAPI) for each treatment. Scale bar, * 10 μm. ***h***, The number of MCT-1 positive Schwann cells (*n* = 4 mice per group). ***i***, Representative images of mitochondrial findings for each treatment. Evaluation was performed in axons and Schwann cells separately. Scale bar, * 200 nm. ***j***, Violin plot of mitochondrial diameter (nm; *n* = 200). Cumulative mitochondria diameter per individual are also shown (*n* = 4 rat per group). ***k***, Quantitative analysis of atypical mitochondria (%; *n* = 4 mice per group). All examinations were performed 2 d (48 h) after administration and evaluated 3 mm distal from the sectional end and the corresponding uninjured nerve. The ROI for measuring ATP levels was set at 3 mm distal from the sectional end, the center of the longitudinal nerve cross-section, with a 300 × 300-μm square (Extended Data Fig. 3-1). The OFP/GFP ratios ranged from 1.5 to 3.0. Immunohistochemistry showed the same results for the four individuals. Error bars indicate SD; *p < 0.05, **p < 0.01, ***p < 0.001, one-way ANOVA followed by the Tukey’s *post hoc* test (for comparison of sham group and transection group, respectively). EP, ethyl pyruvate; MCT-1, monocarboxylate transporters 1; ROI, region of interest.

The results of inhibitor experiments also suggested that the glycolytic system is activated to sustain ATP levels in the distal nerve stump. We sought that supplementing pyruvate would contributes to delay WD through MCT-induced monocarboxylate transport and ATP production via the glycolytic pathway. Then, we tried EP supplementation immediately after axotomy and evaluated delayed WD after 2 d (48 h). Both the low (3 mm) and middle (10 mm) concentrations EP groups showed increased ATP levels compared with the saline group (Fig. 8*a*,*b*). The low (3 mm) concentrations EP group showed the most significant increase in ATP levels compared with the saline group. In contrast, the high (30 and 100 mm) concentrations EP group showed a decrease in ATP levels (data not shown). Both EP groups showed an increase in the number of myelinated axons, a decrease in the G-ratio (Fig. 8*c–f*), and an increase in MCT-1 positive Schwann cells (Fig. 8*g*,*h*). Finally, neither EP group showed improved mitochondrial atypicality in axons and Schwann cells (Fig. 8*i–k*). Together, these findings suggest that the pyruvate contribute to supplying ATP and delaying WD through glycolytic system.

## Discussion

Our study with spatiotemporal *in vivo* ATP imaging showed a gradually decreased levels of ATP in the distal nerve stump. In addition, we determined the metabolic source of ATP in degenerative axons with using mass spectrometry, histologic analysis, and pharmacological intervention. The glycolytic system both in Schwann cells and axons, and MCT-induced monocarboxylate transport were determined as the primary metabolic source of ATP following axotomy, which would delay WD. TCA cycle inactivation, in axons, gradually reduced ATP levels in the distal nerve stump, which indicates and is associated with WD progression.

In the PNS system, Schwann cells supply energy for the maintenance of axons (Beirowski et al., 2014; Nocera and Jacob, 2020; Stassart and Woodhoo, 2020). To evaluate precise ATP levels, ATP levels should be measured in axons and Schwann cells. Previous studies have used luciferase imaging, mass spectrometry, and magnetic resonance spectroscopy to measure intracellular ATP levels (Kennedy et al., 1999; Morikawa et al., 2012; Haris et al., 2014). However, these methods were unable to quantitatively measure ATP levels in real time or within different intracellular compartments. The ATP levels and ATP-related pathologic pathways involved at the distal nerve stump, have been variously reported (J. Wang et al., 2005; Di Stefano et al., 2014; Summers et al., 2014; Gerdts et al., 2015). Therefore, in this study, we used *GO-ATeam2* knock-in mouse and rat models, enabling the evaluation of ATP levels in the cytoplasm of axons and Schwann cells.

We showed that ATP levels 3 mm distal from the sectional end were maintained for 360 min and decreased 2 d later with fluorescence microscopy, and gradually decreased from 1 d later with two-photon microscopy. A previous *in vivo* study showed that the distal nerve stump ATP levels were maintained for over 30 h (Gerdts et al., 2015), this is almost consistent with our findings. However, an in vitro study without Schwann cells showed a rapid decrease in ATP levels (Yang et al., 2015). Our *in vivo* imaging with the *GO-ATeam2* probe demonstrated that the reaction of Schwann cells to energy retention after axotomy contributed to a gradual decrease in ATP levels. We also showed that ATP levels at the sectional end with fluorescence microscopy, decreased immediately after axotomy. This may be because of the initial physical damage with axotomy, since it takes a few hours for the initial response (Carvalho et al., 2019; Hussain et al., 2020). However, toluidine blue staining and electron microscopy revealed WD progression 2 d after axotomy, which was preceded by a decrease in ATP levels of the same lesion, measured by two-photon microscopy, 1 d after axotomy. The correlation between the WD and the ATP decreasing rate is consistent with that observed in previous reports (Beirowski et al., 2005; Yang et al., 2015).

In this study, we showed that the glycolytic system in both Schwann cells and axons is the primary metabolic source of ATP production in the distal nerve stump. Immunohistochemistry detected activation of glycolytic enzymes including HK-1, PFK-1, and LDHA in both Schwann cells and axons, and also detected a decrease in TCA cycle enzyme in axons. Mass spectrometry of the distal nerve stump revealed a decrease in ATP, NAD^+^, and glycolytic intermediates including pyruvate. A previous study revealed that Schwann cells activated glycolysis to provide energy after axotomy by transporting glucose into cells via the glucose transporter 1 (GLUT1) and using glycolytic intermediates (Babetto et al., 2020). We additionally demonstrated the same metabolic changes in axons. The activation of LDHA in both Schwann cells and axons demonstrate the conversion of pyruvate to lactate and NAD^+^ (Doherty and Cleveland, 2013). NAD^+^ is crucial for the production of ATP in glycolysis (Fantin et al., 2006; Diaz-Garcia et al., 2017; Urbanska and Orzechowski, 2019; Osis et al., 2021). This is corroborated by the treatment of 2-DG in the transection side; compared with the DMSO control group, the following was observed: WD progression and decreased levels of ATP without degenerated axonal mitochondria. These results suggested that the glycolytic system played an important role in maintaining ATP levels via NAD^+^ utilization that are generated from the catabolism with LDHA in the distal nerve stump in Schwann cells and axons.

We also found that MCTs in Schwann cells are activated after axotomy, which suggests its relevance in maintaining ATP levels in the distal nerve stump. An increase of MCT-1 positive Schwann cells was detected, using immunohistochemistry, after axotomy. Treatment with 4-CIN also demonstrated a remarkable progression of WD and decreased levels of ATP only in the transection side, and a slight progression of WD and unchanged levels of ATP in the sham side, compared with the DMSO group. Additionally, treatment with 2-DG, MSDC-0160, and 4-CIN demonstrated an increase in MCT-1 positive Schwann cells, compared with that observed in the DMSO group. In the PNS system, both MCT-1 and MCT-4 are expressed in Schwann cells (Garcia et al., 1994; Domènech-Estévez et al., 2015). In uninjured nerve, the absence of MCT-1 and MCT-4 in Schwann cells dose not present developmental disorders of the PNS (Jha et al., 2020; Bouçanova et al., 2021). In contrast, in injured nerve, MCTs are activated to maintain axonal integrity by transporting monocarboxylate from Schwann cells to axons (Babetto et al., 2020). MCTs are also responsible for the passive and bidirectional transport of monocarboxylates such as pyruvate and lactate (Pérez-Escuredo et al., 2016). Given the need for pyruvate in axons, it is probable that pyruvate is transported from Schwann cells toward axons, whereas accumulated lactate in axons is transported from axons to Schwann cells. Additionally, lactate produced or transported by Schwann cells may be transported extracellularly via MCTs, which corroborates previous research that demonstrated extracellular lactate aggregation (Babetto et al., 2020). Our findings suggested that MCTs in Schwann cells are activated in cases of injury or energy metabolic inhibition, to support the activation of the glycolytic system in axons to efficiently maintain ATP levels.

**Figure 9.**
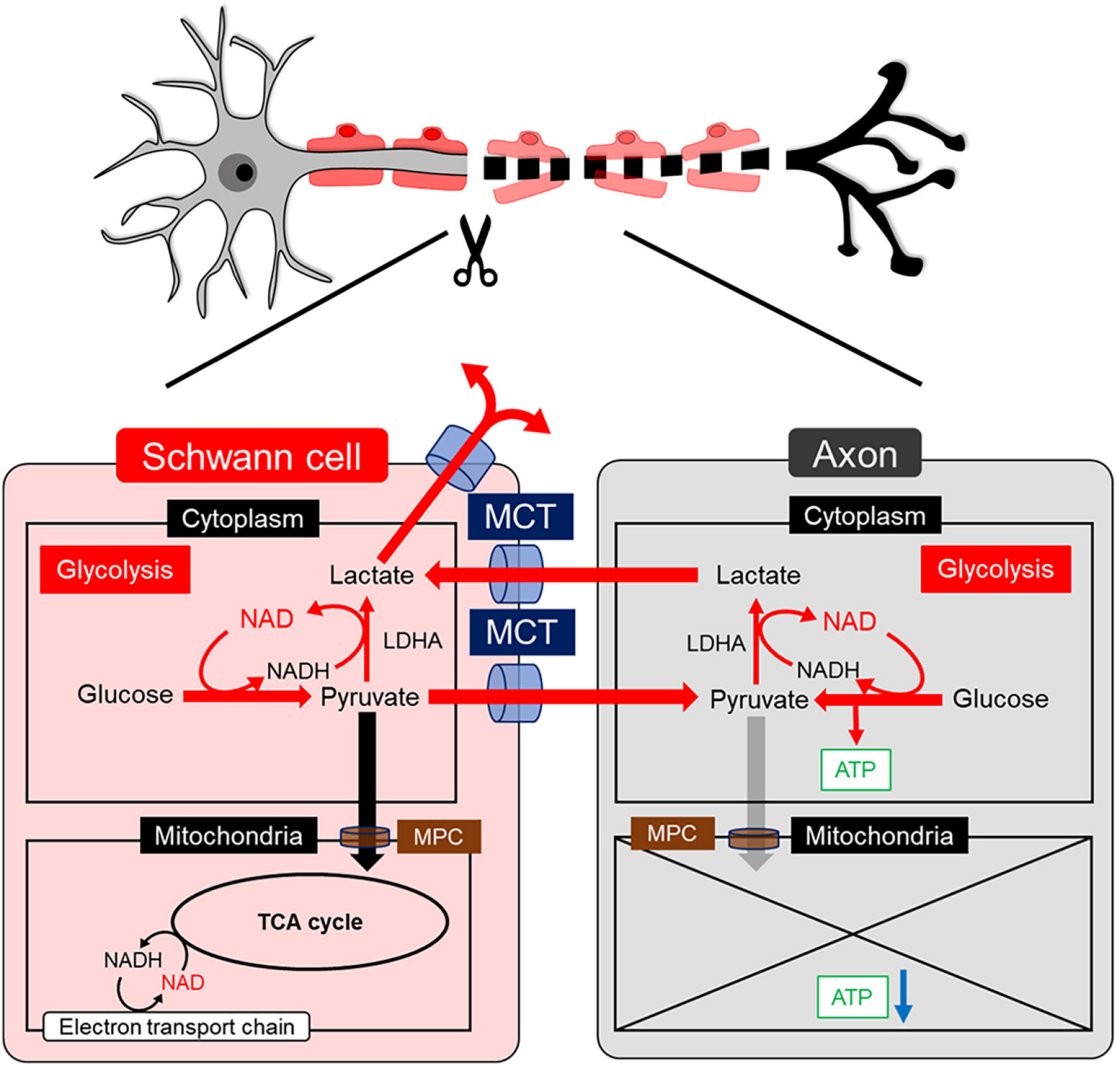
Model depicting changes in the energy metabolism of Schwann cells and axons after axotomy. The proposed metabolic changes in the distal nerve stump. In Schwann cells, the glycolytic system and MCT-induced monocarboxylate transport are activated. In axons, the glycolytic system is activated, whereas TCA cycle is inactivated because of mitochondrial degeneration. The glycolytic intermediate, pyruvate is transported from Schwann cells to axons through MCTs, and both in Schwann cells and axons, pyruvate is metabolized by LDHA to generate NAD^+^ and promote metabolic shifts toward the glycolytic system to produce ATP. The main metabolic source of ATP is mainly on glycolytic system both in Schwann cells and axons. The excess produced lactate in axons is transported to Schwann cells, subsequently released to extracellular space via MCTs.

We demonstrated that EP supplementation resulted in WD delay, increased numbers of MCT-1 positive Schwann cells, and elevated levels of ATP, compared with the saline group. In injured axons, EP supplementation delays WD by increasing ATP levels (J. Wang et al., 2005), inhibiting de-differentiation of Schwann cells through p-ERK1/2, p75NGFR, and lysosomal associated membrane protein 1 (Park et al., 2015), and preventing the expression of neuronal nitric oxide synthase (NOS1; Chung et al., 2019). Among these diverse pharmacological effects of EP, a constructive impact on ATP production was observed. Pyruvate delays WD by inhibiting the activation of poly-ADP ribose polymerase 1 (PARP-1), which results in NAD^+^ depletion and inhibition of glycolysis (Zilberter et al., 2015). In addition, pyruvate contributes to ATP level maintenance through increased glycogen levels (Shetty et al., 2012). The results of EP supplementation experiment suggested that the additional pyruvate may have been transported from Schwann cells to axons via MCTs, thereby augmenting the glycolytic system and increasing ATP levels. EP supplementation concentrations have been reported to vary depending on the experimental system and route of administration (Park et al., 2015; Chung et al., 2019; Chavali et al., 2020; Gaikwad et al., 2021; Mao et al., 2021) thus EP toxicity at high concentrations (100 mm or higher) should be noted (Chung et al., 2019). In the present study, protective effects were observed in low concentrations (3 and 10 mm). The effects of high EP concentrations (30 and 100 mm) observed in this study indicates the toxicity inversely, thus it was desirable to identify the optimal dosage concentration of EP for the nervous system. Together, the present study suggested that to maintain ATP after axotomy, pyruvate is transported from Schwann cells to axons through MCTs, and both in Schwann cells and axons, pyruvate is metabolized by LDHA to generate NAD^+^ and promote metabolic shifts toward the glycolytic system, yet generate inadequate and inefficient ATP levels. We propose the model that the distal nerve stump produces ATP through MCT-induced monocarboxylate transport and the glycolytic system both in Schwann cells and axons (Fig. 9).

We also showed that TCA cycle in axons was not significantly associated with further progression of WD after axotomy. The treatment with MSDC-1060 of the transection side, demonstrated neither progression of WD nor decreased levels of ATP, compared with the DMSO group. In the distal nerve stump, previous studies also showed that mitochondrial disfunction model with carbonyl cyanide 3-chlorophenylhydrazone (CCCP) does not accelerate WD (Loreto et al., 2015). The protective effect of Wallerian degeneration slow (*wld^s^*) protein is independent of mitochondrial function (Kitay et al., 2013), and the contribution of mitochondrial dysfunction to WD progression has no established explanation (Merlini et al., 2022). Despite the inhibition of the glycolytic system, an upstream metabolic pathway to the TCA cycle, 2-DG demonstrated no change of axonal mitochondrial atypicality in the transection side compared with DMSO group. These findings suggest that the TCA cycle may be not significantly associated with the salvage pathway during WD, as a metabolic source of ATP.

Although this study demonstrated biochemical alterations after axotomy, the TCA cycle can also be considered an essential energy metabolic pathway for efficient ATP production. First, in the distal nerve stump, we demonstrated ∼80% axonal mitochondrial atypicality, decreased levels of ATP, and decreased availability of the TCA cycle intermediates and enzymes. That is, ∼20% of the mitochondria in axons are maintained 2 d after axotomy. It was previously reported that calcium overload caused an opening of the mitochondrial permeability transition pore (mPTP) and a decrease in mitochondrial ATP, which lead to WD progression in the distal nerve stump (Villegas et al., 2014). It was also reported that mitochondrial localization of NMNAT activity results in increased ATP synthetic capacity without affecting the expression of the mitochondrial enzymes profile of the respiratory chain (Yahata et al., 2009), and increased NMNAT activity delayed WD (Araki et al., 2004). These findings indicated that the inactivation of TCA cycle is associated with gradually decreased levels of ATP after 1 or 2 d after axotomy, and also suggested that activation of TCA cycle through NMNAT activity may delay WD. The production of ATP by the TCA cycle is not fully inactivated after axotomy and may still persist to a certain degree. Second, we showed that the treatment with MSDC-1060 of the sham side, demonstrated decreased levels of ATP and progression of WD, compared with the DMSO group. The TCA cycle functions in the mitochondrial matrix and requires the influx of pyruvate via the MPC1 and MPC2 to initiate the reaction (Bricker et al., 2012), and the defect of MPC disrupts the homeostasis of glucose metabolism (Sébastien Herzig, 2012; Vigueira et al., 2014). In uninjured axons, mitochondrial dysfunction is associated with WD in uninjured axons; CCCP triggers mitochondrial depolarization and leads to WD (Loreto et al., 2020). Together, these findings suggested that the TCA cycle is responsible for maintaining uninjured nerve stability and its ATP levels, and also may be partially responsible for injured nerve. Further investigations are necessary to elucidate the correlation between the TCA cycle and the ATP-related salvage pathway during WD.

A recent study showed the necessity of the glycolytic system in axons for not only axonal maintenance but also regeneration (Ketschek et al., 2021). The current study focuses on the novel perspective of the glycolytic system rather than mitochondrial function, which has been attracted significant attention in neuronal metabolism (Sheng, 2017). The axons initiated axonal regeneration without migrating Schwann cells in the acute phase, within 5 d of axotomy, which occurred concurrently with WD progression (Min et al., 2021). Glycolytic intermediates support cells involved in axon regeneration, such as macrophages, mesenchymal and endothelial cells (Cattin et al., 2015; Babetto and Beirowski, 2022). Therefore, the activation of the glycolytic system in axons may be a strongly associated with WD delay and axon regeneration. Axonal regeneration and prevention of muscle atrophy following the early neurorrhaphy (Yuan et al., 2019; Raza et al., 2020), may be supported by axonal ATP retention. Additional studies would be needed to clarify the mechanisms of neuronal metabolism with focusing on the glycolytic system.

There are some limitations in this study. First, the local administration experiments of inhibitors and pyruvate were targeted in both Schwann cells and axons. ATP levels were also targeted at the center of the longitudinal nerve cross-section even in two-photon microscopy imaging. The Schwann cells and axons could not be completely separated. In addition, Schwann cells make up ∼45–70% of the cellular components in the peripheral nerve (Stierli et al., 2018; Yim et al., 2022), and interference with ATP levels by macrophages and other constituent cells such as endoneurial fibroblasts, adipocytes, and pericytes, should be considered (Stierli et al., 2018; Stassart and Woodhoo, 2020; Yim et al., 2022). These problems would be solved by using Schwann cell-specific (P0-Cre) and axon-specific (NFH-Cre) *GO-ATeam2* knock-in mice (Feltri et al., 1999; Scemes et al., 2019). Alternatively, we showed the activation of the glycolytic system and TCA cycle in Schwann cells and in axons by immunohistochemistry. Second, the present study did not thoroughly analyze the role of the TCA cycle in the progression of WD. Previous studies suggested that the TCA cycle is associated with active processes in WD. The TCA cycle produces ATP via autophagic activity (Lum et al., 2005). Autophagy is triggered by glycogen synthase kinase 3B (GSK3B)-induced activation of mitochondrial MCL1, and contributes to local ATP in degenerating axons, which is required for normal recruitment of phagocytes to axonal debris *in vivo* (Wakatsuki et al., 2017). In this study, only infiltrated CD68 macrophages were detected after axotomy, and its metabolic interaction was not investigated. We should note that the TCA cycle is also involved in the ATP-related auto-destruction programs during WD, as well as in the salvage pathway. Furthermore, the treatment with MSDC-0160 in the transection side did not demonstrate decreased levels of ATP or progression of WD as compared with the DMSO group. The adjuvant effect of pharmacological treatment may be relatively modest considering the spontaneous axonal mitochondrial degeneration in the distal nerve stumps. Finally, the potential effects of MCTs on Schwann cells remain unclear (Jha et al., 2020). Cell-specific experimental methods are required to support the results reported herein.

Our study expanded the understanding of spatiotemporal ATP levels in injured peripheral nerve and the dynamics of energy metabolism in WD. Our findings suggest a metabolic shift toward the glycolytic system for maintaining ATP levels, both in Schwann cells and in axons. This study provides a basis for understanding the bioenergetics during WD, and could be used in the development of therapeutic agents.

## Synthesis

Reviewing Editor: Tod E Kippin, University of California Berkeley Decisions are customarily a result of the Reviewing Editor and the peer reviewers coming together and discussing their recommendations until a consensus is reached. When revisions are invited, a fact-based synthesis statement explaining their decision and outlining what is needed to prepare a revision will be listed below. The following reviewer(s) agreed to reveal their identity: Simone Di Giovanni. Note: If this manuscript was transferred from JNeurosci and a decision was made to accept the manuscript without peer review, a brief statement to this effect will instead be what is listed below.

I have now received two independent reviews of your manuscript and they concur that it is still not suitable for publication in eNeuro. Although the manuscript is much improved, there are still major issues. In particular, I agree with Reviewer 1 that the Introduction still requires substantial editing in order to provide a clear and concise rationale for the study-as indicated some of the responses in the Author’s rebuttal are much clearer than the text for the manuscript and need to be incorporated into the manuscript. Many of these issues were raised during the review of the initial manuscript and have not been adequately addressed in the revision. Please ensure that all concerns appropriately dealt with prior to resubmission.

Below are comments from the Independent Reviewers:

Reviewer 1

This is a revised manuscript. In the previous round of review, no additional experiments were requested, but it was identified that the motivation of the study was poorly described. Authors addressed the raised concerns mainly with revised and additional texts and revised statistical analyses. Frankly, while I can see and appreciate the efforts, the manuscript is still a very difficult read. To me, the first sentence in the Abstract, “Axonal injury causes Wallerian degeneration (WD) in the distal nerve stump, but adenosine triphosphate (ATP) behavior remains unclear” exemplifies my concern for the lack of explicitly expressed scientific rationale that is seen throughout the manuscript. However, I feel that offering thorough feedback on writing itself is beyond my role as a reviewer. I will make one major comment on it and leave it in the hands of the editors and will mainly focus on the scientific rigor and interpretation. My specific comments are below:

1) The motivation of the study provided in Response 1-1 adequately addressed my previous concern, however, in the main text, one won’t get to it until the third paragraph. In fact, the following main bullet points are all there in the Introduction, but they are quite scattered across the four paragraphs, making the rationale difficult to access:

• WD is programmed axon degeneration (paragraph1)

• Elevation of axonal ATP, as an essential substance for energy metabolism in the nervous system, is implicated in delaying WD. (paragraph 3)

• Activation of the glycolytic system in Schwann cells provides energy support in the form of monocarboxylate to injured axons and delays WD (paragraph 3)

• Metabolic pathways utilizing these monocarboxylates (glycolysis vs TCA cycle) to generate ATPs in injured axons is unclear (paragraphs 3 and 4)

2) Response 1-2 clarifies “Previous studies did not clarify how monocarboxylate transported to the axon are metabolized in injured axons”. I agree that this is the key take-home from this study in addition to identifying the role of glycolysis in the injured axons. However, what is not clearly discussed is the link between the monocarboxylate transport from Schwann cells to the injured axon and the direct impact on ATP production. In this study, the authors claim that the TCA cycle play no role in ATP production in the injured axon, which is the key difference in the model between this study and Babetto et al., 2020 (also cited in this study), which hypothesized that the monocarboxylates (pyruvate and lactate) transported from Schwann cells into the injured axon will promote ATP production via TCA cycle (although the latter portion of this hypothesis was not directly tested). Authors arrived at this interpretation because MSDC-0160 did not further reduce ATP levels in injured axons while 2-DG and 4-CIN did. If TCA indeed plays no role in ATP production in the injured axons, then are we to understand that the transported monocarboxylates are merely supporting the axonal glycolysis by providing NAD+? I think the authors touch on it on page 20 when they state “The metabolic changes in our study suggested that pyruvate and NADH converted to lactate and NAD+ (...), subsequently GA3P and NAD+ converted to PEP”, however, this is buried in confusing messages like “...activation of the glycolytic system both in Schwann cells and axons via MCT-induced monoccarboxylate transport supply axonal ATP levels” (pg19).

3) According to Fig7l, it appears that ∼30% of the mitochondria in injured axons are still maintaining normal appearance. I find it difficult to unequivocally conclude that they are not supplying ATPs, especially since there appears to be a trend in ATP reduction in MSDC-0160 group in Fig7b. MSDC-0160 indeed does strongly affect ATP levels acutely after the axonal injury before the onset of mitochondrial degeneration (Fig.7-1). Due to the already reduced level of ATP in the transected nerves in DMSO group (presumably because ∼70% of the mitochondria have degenerated), any additional effects of the pharmacological treatment are expected to be modest. n=6 may not confer a very strong statistical power in this case. I would encourage the authors to possibly tone down their conclusion that TCA cycles plays no role in supplying ATPs in injured axons.

Minor comments:

1) Identification of the ROIs as indicated in Fig.1-1 seems rather crude. Were there any physical markers other than the edge of the transected nerves that were used to assure that same ROIs were identified across the 14d chronic imaging period (Fig1c)? With the edges possibly deteriorating over time, simply using the edge as the marker may result in the shifting of ROIs over time.

2) It appears that the images of proximal and distal portions of the severed nerve shown in Fig.1C were contrast or brightness adjusted independently from each other. Since quantifications are done with ratiometrically, it is unlikely to greatly impact the measurements, but it is still a good practice to keep background conditions as similar as possible.

3) Fig. 7k lacks statistical analyses.

Reviewer 2:

Numerous studies have been published providing essential, yet incomplete understanding of the mechanisms of Wallerian degeneration (WD). Takenaka et.al addressed this issue from a metabolic perspective. They concluded that ATP decreased during WD progression and identified the glycolytic system as the major source of ATP retention by using ATP imaging, LC-MS, histological analysis and pharmacological manipulation.

This article is well-written, and the results are well supported by a variety of methods. Authors used the GO-ATeam2 knock-in rodents for ATP imaging which allowed a spatiotemporal view of ATP during WD progression. And they also applied two-photon microscopy to eliminate the effect from epineurium or fibrofatty tissue.

There are still some issues that the authors shall pay attention to and address more explicitly.

major issues:

• The diagram in figure 9 shall be revised and the authors should discuss the directionality of monocarboxylate transporters (MCTs) from schwann cells to axons. The authors mentioned this in the discussion (line 547), but by reading the two references, the data remain not fully conclusive under injury conditions. As far as we know, MCTs facilitate the passive and bidirectional transport of monocarboxylates such as lactate and pyruvate (Perez-Escuredo et al., 2016). Because of the following reasons, the direction seems to be opposite to the diagram you showed in figure 9.

1. In figure 9, it seems that the pyruvate or lactate has lower concentration in schwann cells, due to (1) the consumption of pyruvate by TCA cycle, (2) the export of lactate, (3) the activation of LDHA; however pyruvate or lactate in injured axons should accumulate due to the activation of glycolysis and the malfunction of mitochondria.

2. In figure 5&6, authors stated that after axonal injury, the glycolysis is upregulated, and the TCA cycle was inactivated in neurons while in schwann cells glycolysis was activated.

3. Further, as authors showed in figure 2 e-g, the alteration of mitochondria in axons occurred in an earlier time-point compared to schwann cells, which may indicate that the TCA cycle impairment and accumulation of pyruvate in neurons precede the one in schwann cells.

Could the authors discuss more in detail about the direction of the pyruvate from schwann cells to axons? Is it possible to provide some evidence of the accumulation of pyruvate in schwann cells 48h post-injury?

• The experiment in figure 8 may not support the conclusions. The authors pointed out that the supplemented ethyl pyruvate could contribute to glycolytic activation, resulting in the delay of WD by measuring the ATP level, the number of myelinated axons and mitochondria morphology.

However:

1. Ethyl pyruvate and methyl pyruvate has been reported to increase ATP level and show positive effect of inhibiting axonal degeneration by other pathways: inhibiting Nitric Oxide Synthase (Chung et al., 2019), inhibiting p-ERK1/2, p75NGFR, and LAMP1(Chung et al., 2019); increasing ATP (Park et al., 2013)

2. The accumulation of ATP does not necessarily represent the activation of glycolysis, since ATP is consumed by loads of processes.

As a result, the authors may need to provide more evidence of the activation of glycolysis in neurons either by performing LC-MS (especially the concentration of lactate and NAD+) or at least immunohistochemistry for the glycolytic proteins (especially LDHA and LDHB).

minor issues:

• In method, line 281, mass spectrometry is not abbreviated to LC-MS. Moreover, the description of method for LC-MS is not detailed enough. Could the authors provide more information on tissue processing, ionization method, column, cycle, data processing and normalization method, etc?

• For all the correlation plots (figure 2d, figure 7f, figure 7g, figure8f), could the authors provide the correlation coefficient, p value and R^2?

• In figure 4, line 384 said “... alpha-ketoglutaric acid, showed non-significant decrease after axotomy”, but figure 4 lower square showed significant decrease of alpha-ketoglutaric acid.

• In figure 4, the glycolysis data from LC-MS showed significant increase in metabolites, but the authors stated that these results suggested the activation of glycolytic pathway (line 381). Could authors explain?

Refs

Chung, H. J., Kim, M., Jung, J. & Jeong, N. Y. (2019). Inhibition of Neuronal Nitric Oxide Synthase by Ethyl Pyruvate in Schwann Cells Protects Against Peripheral Nerve Degeneration. Neurochem Res, 44, 1964-1976.

Park, J. Y., Jang, S. Y., Shin, Y. K., Koh, H., Suh, D. J., Shinji, T., Araki, T. & Park, H. T. (2013). Mitochondrial swelling and microtubule depolymerization are associated with energy depletion in axon degeneration. Neuroscience, 238, 258-69.

Perez-Escuredo, J., Van Hee, V. F., Sboarina, M., Falces, J., Payen, V. L., Pellerin, L. & Sonveaux, P. (2016). Monocarboxylate transporters in the brain and in cancer. Biochim Biophys Acta, 1863, 2481-97.

## Author Response

Reviewer #1

Major comments

Comment 1-1:

It is my understanding that Wallerian degeneration (WD) is an important key step after traumatic nerve injury that will set the stage for potentially successful functional recovery through axonal regeneration and reinnervation of target tissues. It appears that the aim of this study is to determine how one can influence the progression of WD with the focus on ATP supply but none of that is explained in the current manuscript. In fact, it took me a several reads to note that the point of the paper may be why it takes the severed nerve distal to injury a day or two to be depleted of ATP in vivo when injured axons in in vitro setting without Schwann cells show a more rapid decrease in ATP levels. Since pharmacological manipulation of glycolysis and thereby faster reduction in ATP 2 in the distal nerve stump accelerates WD, I’m inclined to think that the aim of the study may be to locate the source of ATP so that one can negatively manipulate it in order to promote WD progression and clear the stage for axonal sprouting from the proximal end. Again, this is missing from the manuscript and it is up to the authors to effectively motivate their study.

Response 1-1:

We agree with this comment and apologize the inadequate description. The elevation of ATP levels in axon delays WD (Wang et al., 2005; Yang et al., 2015) and supports axon regeneration (Han et al., 2016; Zhou et al., 2016). The glycolytic system in Schwann cells is important for delaying WD and axon regeneration (Li et al., 2020; Trimarco and Taveggia, 2020), and monocarboxylate transporters (MCTs) in Schwann cells activate to support injured axons (Babetto et al., 2020), suggesting metabolic interaction between Schwann cells and axons (Boucanova and Chrast, 2020). However, as you pointed out, the spatiotemporal distribution of ATP and the metabolic source of ATP after axotomy has not been studied sufficiently. Therefore, we analyzed the spatiotemporal ATP levels using in vivo intracellular ATP imaging, and tried to clarify metabolic changes on WD progression.

Our findings suggest that glycolytic system is the main source to retain axonal ATP levels, and delays WD in the distal nerve stump. With regard to the above mentioned, we revised the

Introduction section to appropriately inform the motivation for this research. In discussion section, we described that the early neurorrhaphy in addition to the retention axonal ATP levels in distal nerve stump may support nerve regeneration and reduce muscle atrophy. (Yuan et al., 2019; Raza et al., 2020).

Comment 1-2: 3

The main finding reported in this study is that ATP levels are maintained in the distal nerve stump for some time due to glycolysis using the monocarboxylates transported from the Schwann cells into the distal axons. TCA cycle does not play a significant role in providing ATPs. However, many components of this finding have already been reported elsewhere. 1) the role of the glycolytic system in Schwann cells and MCTs in providing energy substrate to distal nerve stump (Babetto et al., 2020), 2) Glycolysis in the cytosol and TCA cycle in the mitochondria are the two suppliers of ATP, and 3) mitochondria is degenerated early in WD (Wang et al., 2021) and that morphologically degenerated mitochondria is incapable of ATP production (Pivovarova and Andrews, 2010). Thus, my enthusiasm towards the impact of this study is waned.

Response 1-2:

We sincerely appreciate your thoughtful comments. Previous studies did not clarify how monocarboxylate transported to the axon are metabolized in injured axons. Although the present study is an indirect assessment of ATP levels, immunohistochemistry, and the pharmacological intervention, we presented that the glycolytic system is activated in axon to maintain ATP levels and to delay WD. We revised the Discussion section below. ATP supplementation through the glycolytic system in Schwann cells has been suggested to be an important role during WD (Babetto et al., 2020; Trimarco and Taveggia, 2020). In addition to these findings, the present study showed that axons also activate the glycolytic system. In injured axons, the supplemented pyruvate would contribute to glycolytic activation. The conversion of pyruvate to lactate is catalyzed by LDHA, with activation of the glycolytic system, resulting in increased NAD production (Fantin et al., 2006; Urbanska and Orzechowski, 2019; Osis et al., 2021). Given our results showing the shift of lactate dehydrogenase activity toward LDHA rather than LDHB both in Schwann cells and in axons after axotomy, we propose that the distal nerve stump demands an early cycle axonal ATP retention system with Schwann cells through MCT-induced monocarboxylate 4 transport and the glycolytic system in axons (Figure 9). A recent study also showed the necessity of the glycolytic system in axons for not only axonal maintenance but also regeneration (Ketschek et al., 2021). Focusing on the glycolytic system is a new perspective that differ from mitochondrial function, which has been attracted in neuronal metabolism (Sheng, 2017). The axons themselves initiate axonal regeneration without migrating Schwann cells in the acute phase within 5 days after axotomy, which is overlapped with WD progression (Min et al., 2021). Therefore, the activation of the glycolytic system in axons may be a strongly associated process not only in delaying WD but also in regenerating axons. Axonal regeneration and prevention of muscle atrophy following the early neurorrhaphy (Yuan et al., 2019; Raza et al., 2020), may be strengthened by axonal ATP retention. Additional studies would be needed to clarify the mechanisms of neuronal metabolism with focusing on the glycolytic system.

Comment 1-3:

Pharmacological intervention of the glycolysis and TCA cycle is used to differentiate the relative contribution of each system to ATP supply. The authors state “...only on the same side of the 2-DG group show a progression of mitochondrial atypicality...., the transection side ... did not show progression of mitochondrial atypicality...”. This, to me, is a very misleading interpretation of the data that solely relies on the statistical significance of the difference between the control (DMSO) and 2-DG while ignoring the fact that the mitochondrial atypicality is already significantly increased in DMSO group on the transection side. The same concern is raised for the effect of MSDC-0160 on ATP levels as the ATP levels are already reduced in DMSO group. MCT + Swchwann cell counts harbors the similar problem when interpreting the 2-DG results as well.

Response 1-3:

We agree with this comment and apologize the inappropriate statistical analysis. In the present manuscript, we ignore the interaction because we adopted one-way ANOVA for statistical analysis. 5

To clarify interaction between the effects of inhibitor treatment and transection, we revised the statistical analysis to two-way ANOVA. Additional statistical analysis methods were written in the

Materials and Methods section. We also wrote the corrections for the analyzed data in the Results section.

Minor comments:

Comment 1-4:

The extended data Figure 1-1 and main Figure 1 appear to have different length of 1mm scale bar relative to the sample.

Response 1-4:

We agree with this comment and apologize the inadequate figures. We corrected the length of the 1 mm scale bar in each figure.

Comment 1-5:

Throughout the text, the authors use the term “suppress” when they refer to the reduction in TCA cycle activity. This is a misnomer as it gives a false impression that it is an active process when it could very well be the direct result of mitochondria degeneration.

Response 1-5:

We agree with this comment and apologize the use of misleading terminology. We revised the part that used “suppress” to “inactivate”. 6 Editor’s Comments:

Summary: The present study explores metabolic changes associated with degeneration of transected peripheral nerves. The study incorporates optical imaging of ATP with extensive histological and molecular analyses as well as includes causal experiments with pharmacological manipulations. Overall, the manuscript is generally well-written and describes some novel findings. Concerns from the manuscript arise from several sources, including potential issues in design/lack of clarity or specific details in methodology, and potentially limited robustness and specificity of some of the effects.

Generally, the overall rationale for the study appears to be somewhat lacking. It is unclear why determining ATP dynamics and their sources is important for transected nerve conditions. The authors allude to developing therapeutic agents based on a greater understanding of the pathology but it is unclear what desired metabolic states would promote (presumably) nerve repair. Overall, the manuscript appears to be poorly matched to a general neuroscience journal in its present form.

Response: We agree with this comment and apologize the inadequate description. ATP is essential for the neuronal metabolism, because it contributes to delay WD and to proceed axon regeneration.

Therefore, we performed in vivo ATP imaging and investigated the biochemical pathways during WD, to determine whether the glycolytic system or the TCA cycle is activated to retain ATP levels.

With regard to the above mentioned, we revised the Introduction section. Furthermore, we added below in the Discussion section, why activating glycolytic system in axon and increasing ATP levels are important for neuronal metabolism. ATP supplementation through the glycolytic system in Schwann cells has been suggested to be an important role during WD (Babetto et al., 2020; Trimarco and Taveggia, 2020). In addition to these 7 findings, the present study showed that axons also activate the glycolytic system. In injured axons, the supplemented pyruvate would contribute to glycolytic activation. The conversion of pyruvate to lactate is catalyzed by LDHA, with activation of the glycolytic system, resulting in increased NAD production (Fantin et al., 2006; Urbanska and Orzechowski, 2019; Osis et al., 2021). Given our results showing the shift of lactate dehydrogenase activity toward LDHA rather than LDHB both in

Schwann cells and in axons after axotomy, we propose that the distal nerve stump demands an early cycle axonal ATP retention system with Schwann cells through MCT-induced monocarboxylate transport and the glycolytic system in axons (Figure 9). A recent study also showed the necessity of the glycolytic system in axons for not only axonal maintenance but also regeneration (Ketschek et al., 2021). Focusing on the glycolytic system is a new perspective that differ from mitochondrial function, which has been attracted in neuronal metabolism (Sheng, 2017). The axons themselves initiate axonal regeneration without migrating Schwann cells in the acute phase within 5 days after axotomy, which is overlapped with WD progression (Min et al., 2021). Therefore, the activation of the glycolytic system in axons may be a strongly associated process not only in delaying WD but also in regenerating axons. Axonal regeneration and prevention of muscle atrophy following the early neurorrhaphy (Yuan et al., 2019; Raza et al., 2020), may be strengthened by axonal ATP retention. Additional studies would be needed to clarify the mechanisms of neuronal metabolism with focusing on the glycolytic system.

Comment 2-1:

Please confirm that each data point in the graphs (e.g. Fig 1b and c) and used for the ANOVA was based on a single image; i.e. that there was no averaging across images to get each data point. If multiple images were used for each data point then please describe how data was organized and analyzed. Similarly, if there was any image selection (from multiple options) then please describe the process. The method indicates how individual images were captured with the implication that only 8 one image was used for each time point (at least to my reading) so I just want to ensure that I am not missing something about the data collection parameters and that these parameters are precisely described.

Response 2-1:

We sincerely appreciate your comment and apologize the inadequate description. The same individuals were used for until 360 min (Figure 1a-b) after axotomy, and the separate individuals were used for until 14 days after axotomy (Figure 1c-d). The guidelines on the rearing period (up to 24 hours) outside of animal laboratory made these differences, since in our institute, the animal laboratory and microscopes are in different locations. Then, we revised the statistical analysis to two-way ANOVA. We revised the manuscript in the Materials and Methods section, and Figure legends.

Comment 2-2:

Some of the statistical analyses may not be optimal and some justification for the selected tests appears warranted. For figure 1b, it would seem a two-way ANOVA (i.e. position X time effects) would be more appropriate than a series of one-way ANOVAs as the latter artificially inflated alpha values. Why was a two-way test not employed? Similarly, for figure 1c, a two-way ANOVA appears more appropriate than an on-way. Lack of correct (two-way ANOVA) statistics are of equal concern in the pharmacological experiments. Either the appropriate statistics should be employed or the selected ones justified.

Response 2-2:

We agree with this comment and apologize the inappropriate statistical analysis. In the present manuscript, we did not pay attention to the interaction between inhibitor treatment and transection. 9

We revised the statistical analysis to two-way ANOVA for both Figure 1 and 7, which were affected from two different factors. Additional statistical analysis methods were written in the Materials and

Methods section. We also wrote the corrections for the analyzed data in the Results section, and revised Figure 1 and 7.

Comment 2-3:

It is unclear why some experiments were performed in rat and other in mouse; it would be helpful to have an explicit (but brief) explanation for why different species were used.

Response 2-3:

We sincerely appreciate your comment. We added the reason for using different species as below in the Materials and Methods section. In the earlier part of the experiment, we used rats, which are more suitable for modeling human disorders and easier to surgically manipulate (Adalbert et al., 2005). As the experiment proceeds, we used mice because the experiments involving two-photon microscopy were unsuitable for the body size of rats.

Comment 2-4:

There appears to be lack of robust effects on some measures. In comparing Fig 2 and Fig 7 the mitochondria diameter is elevated following transection in the former but this effect appears to be lost in Fig 7k. Similarly, the ATP reduction and G-ratio shows a much smaller decrease in the experiment with the inhibitors than in the initial characterization (Fig 2). Are these expected levels of variation in these endpoints across experiments? Is there an explanation for this diminished impact of transection? 10

Response 2-4:

We sincerely appreciate your thoughtful suggestions and apologize the inappropriate description and figures. The species of animals and evaluated each time point as follows: Figure 2: rats, before and one, two, 14 days after axotomy. Figure 3: mice, before and one, two, 7 days after axotomy. Figure 7: mice, before (sham side) and one day (transection side) after axotomy. Figure 8: mice, two days after axotomy. Although the animal species are different, the myelinated axons, G-ratio, and mitochondrial findings at 1 day after axotomy in Figure 2 are almost identical to the transection side (one day after axotomy) of DMSO group in Figure 7. The ATP levels at one day after axotomy in Figure 3 almost correspond to the transection side of the DMSO group in Figure 7. The myelinated axons, G-ratio, and mitochondrial findings at 2 days after axotomy in Figure 2 are almost identical to those of the Saline group (2 days after axotomy) in Figure 8. We added time notations to Figures 7 and 8 to avoid misleading the reader about the evaluated endpoints.

Comment 2-5:

Although the pharmacological inhibition experiments were done in transected as well as sham conditions, the supplementation experiment was not. Given the effects of MCT inhibition in the absence of transection (Figure 4), why were the effects of monocarboxylate supplementation not examined in the absence of transection? It is hard to determine if observed effects are specific to transected structures or not. Has this experiment been reported elsewhere? In general, there were a number of nonspecific effects of the drugs and they are not well discussed. For instance, could these drug manipulations, if used as therapeutics for transected nerves, have negative impacts for intact nerves? More consideration of the entirety of the data may be necessary. 11

Response 2-5:

We sincerely appreciate your thoughtful suggestions and apologize the inappropriate description.

Previous studies showed that EP supplementation delay WD, and in vivo dosing concentrations have been reported (Park et al., 2015; Chung et al., 2019). We showed an increase in ATP levels for low concentrations (3 and 10 mM) of EP, and a decrease for high concentrations (30 and 100 mM). A previous study also showed that local administration of 100 mM or more concentrated EP induced toxic effects (Chung et al., 2019). Therefore, although the effects of EP on uninjured axons have not been reported, we determined that performing only transection group (2 days after axotomy) is sufficient to confirm concentrations of EP toxicity, in terms of minimizing surgical invasions in animals. However, as you point out, the optimal concentration of EP for neuronal metabolism has not been clarified. We added these insights and concerns to the below in the Discussion section. Previous reports have shown that EP delays WD (Park et al., 2015; Chung et al., 2019). We used EP and showed delayed WD and ATP supply without improving mitochondrial atypicality in axons. EP treatment also increased MCT1-positive Schwann cells, suggesting an effect following supplementation of energy substrates. The contribution of pyruvate, a glycolytic intermediate, to the neuronal metabolism has previously been reported. To antagonize WD, pyruvate abates the activation of Poly-ADP ribose polymerase 1 (PARP-1), which causes depletion of NAD and inhibition of glycolysis (Zilberter et al., 2015). and contributes to the retention of ATP levels through increased glycogen levels (Shetty et al., 2012). Glycolytic intermediates also support cells involved in axon regeneration, such as macrophages, mesenchymal and endothelial cells (Cattin et al., 2015; Babetto and Beirowski, 2022). Considering our findings as well, it is suggested that EP supplementation support energy metabolism via increased ATP levels in the distal nerve stump.

Although we reversely showed a decrease in ATP levels for high concentrations (30 and 100 mM) of EP, previous study also showed that local administration of 100 mM or more concentrated EP induced toxic effects in Schwann cells (Chung et al., 2019). High concentrations of EP may be toxic 12 to Schwann cells, resulting in lower ATP levels. Depending on the experimental system and administration route, previous studies reported varying concentrations of EP supplementation (Park et al., 2015; Chung et al., 2019; Chavali et al., 2020; Gaikwad et al., 2021; Mao et al., 2021). To ensure the effectiveness of supplementation against neural metabolism, it is desirable to identify the appropriate dosage concentration of EP for the nervous system.

Minor concerns:

Comment 2-6:

The methods include description of pyruvate manipulations but findings from such experiments do not appear to be described in the results.

Response 2-6:

We sincerely appreciate your thoughtful suggestions. In the present manuscript, In the section on ethyl pyruvate (EP) administration, the words “EP”, “pyruvate”, and “monocarboxylate” are mixed up and confusing the reader. Therefore, we revised and unified the terminology in the Materials and

Methods, Results, and Discussion sections to be “EP”.

Comment 2-7:

Line 489-90: “On the transection side, 2-DG treatment did not result in mitochondrial atypicality in axons”; should be “...did not result in further...” as the transection already increased the atypicality and thus may represent a ceiling effect on this measure.

Response 2-7: 13

We agree with this comment and apologize the use of misleading terminology. We add “further” in the manuscript parts that mention about the transection side.

Comment 2-8: For 7-1-c-d “The concentration of inhibitors was determined according to the change in ATP levels using a fluorescence microscope and measured intermittently for 120 min after axotomy in the same individuals (Figure 7-1c-d). “This is unclear/confusing; I believe they are trying to determine the effectiveness of given concentrations rather than the concentration of the inhibitors.

Response 2-8: We agree with this comment and apologize the inadequate description. We revised as below in the

Materials and Methods section.

Before proceeding to the main experiment, we preliminarily measured the change of ATP levels in the distal nerve stump for 120 minutes intermittently, by using two concentrations of all inhibitors (Figure 7-1c-d). The concentrations of inhibitors were determined from previous studies (Press and Milbrandt, 2008; Miranda-Goncalves et al., 2013; Tsuyama et al., 2013; Patterson et al., 2014; Anamitra Ghosh, 2016; Ichihara et al., 2017; Babetto et al., 2020). Then, we used higher concentrations for all inhibitors in the main experiments.

Thank you very much for your carefully reviewing.

We appreciate for Editors/Reviewers’ warm work earnestly, and hope that the correction will meet with approval.
