# Supplementary figures and images for: Glycolytic System in Axons Supplement Decreased ATP Levels after Axotomy of the Peripheral Nerve
Source: eNeuro. 2023 Mar 17;10(3):ENEURO.0353-22.2023. doi: 10.1523/ENEURO.0353-22.2023 (PMC10035771; doi:10.1523/ENEURO.0353-22.2023)

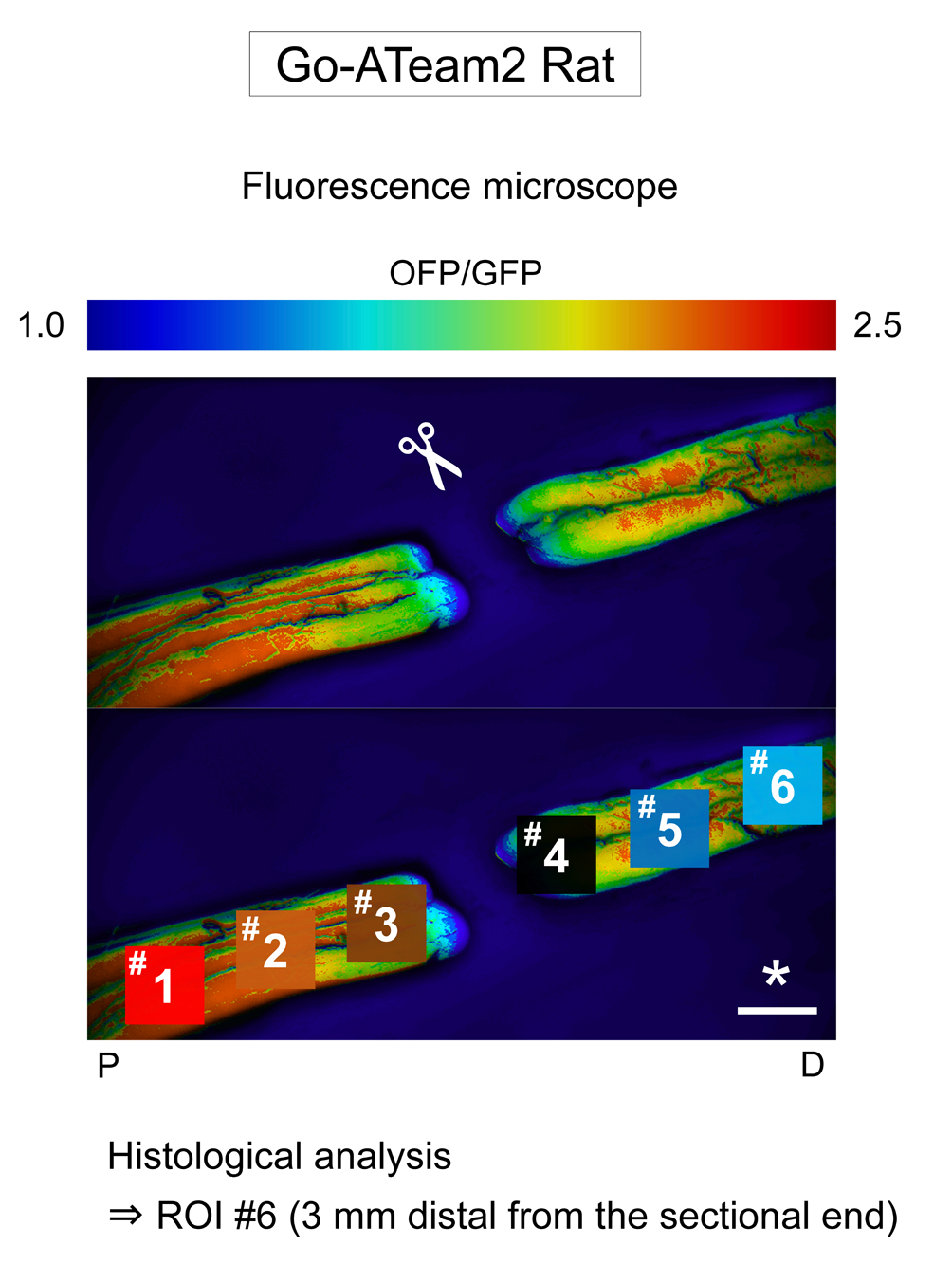

Supplement: Extended Data Figure 1-1 — Experimental system for ATP levels with fluorescence microscopy and histological analysis in rats. A schematic illustration of whole nerve ATP levels measurement for GO-ATeam2 rat. All of fluorescence emission in the GO-ATeam2 probe was captured with fluorescence microscopy. ATP levels were intermittently measured in six ROIs (from ROI #1 to #6), three proximal and three distal sites for axotomy, 1-mm square with 500-μm spacing in the same individual. OFP/GFP ratio ranges were from 1.0 to 2.5. The histological analysis was performed 3 mm distal from the sectional end, correspond to ROI #6. Scale bar, * 1 mm,** 1-mm square ROI. P, proximal. D, distal. Download Figure 1-1, TIF file. [file enu-eN-CFN-0353-22-s01.tif]

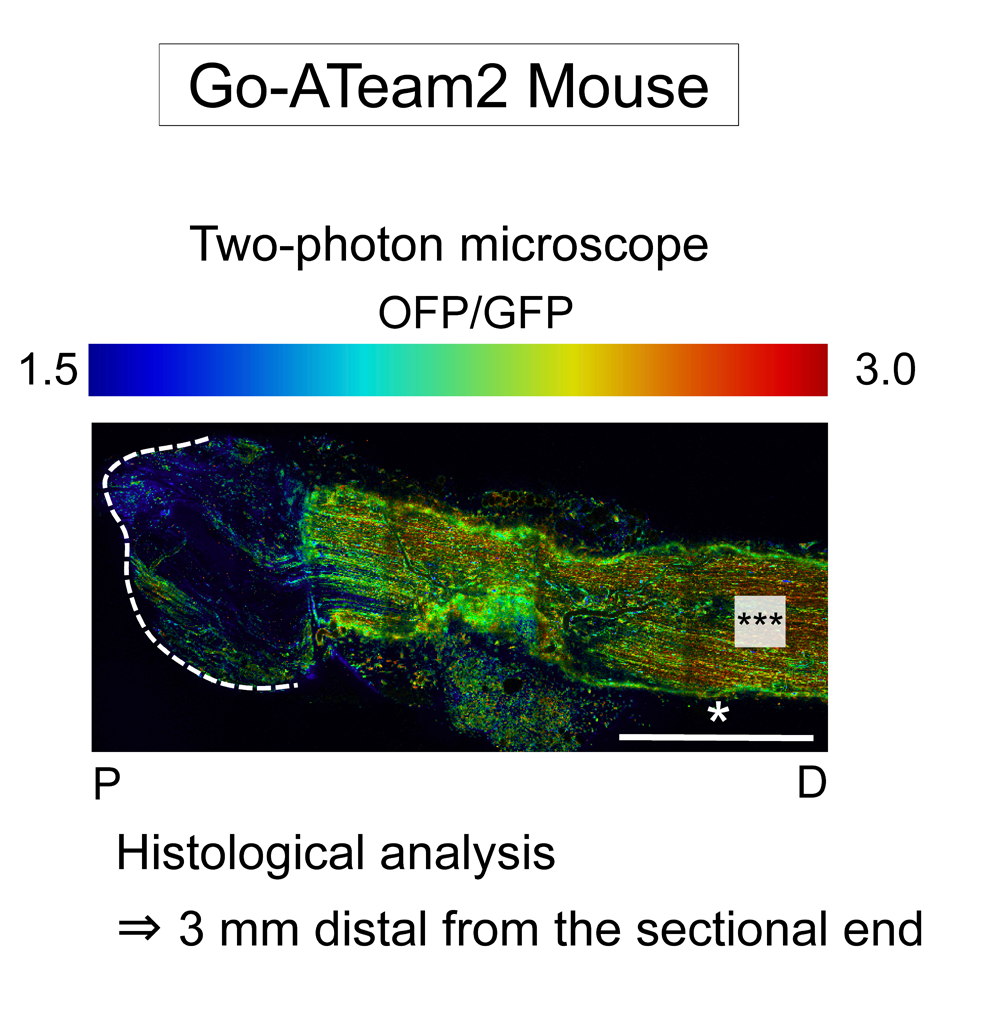

Supplement: Extended Data Figure 3-1 — Experimental system for ATP levels with two-photon microscopy and histological analysis in mice. A schematic illustration of center of the longitudinal nerve cross-section ATP levels measurement for GO-ATeam2 mouse. All of fluorescence emission in the GO-ATeam2 probe was captured with two-photon microscopy. ATP levels were measured in each ROIs (*** 300-μm square), 3 mm distal from the sectional end (broken line). OFP/GFP ratio ranges were from 1.5 to 3.0. The histological analysis was performed 3 mm distal from the sectional end. Scale bar, * 1 mm;*** 500-μm square ROI. P, proximal. D, distal. Download Figure 3-1, TIF file. [file enu-eN-CFN-0353-22-s02.tif]

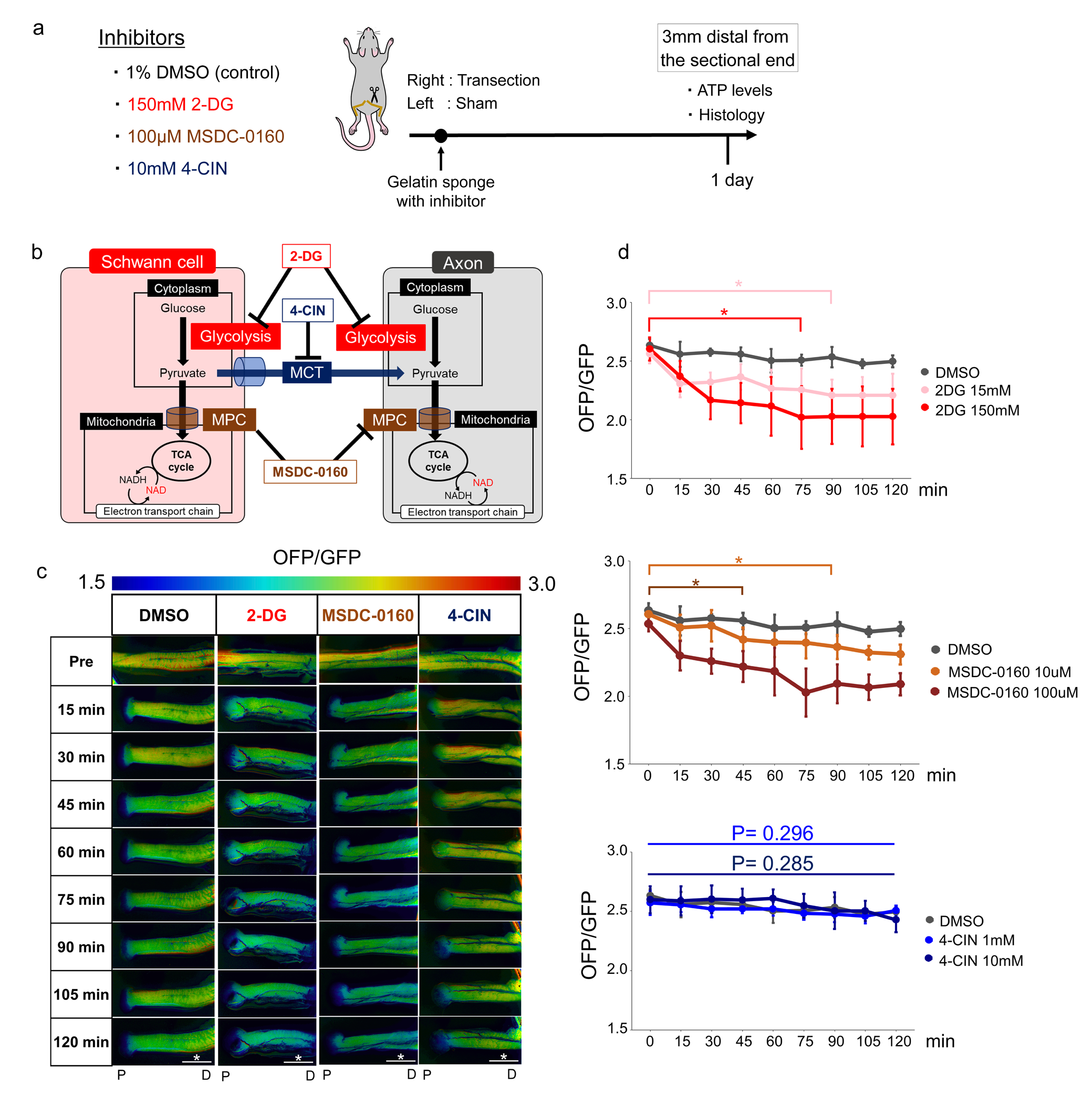

Supplement: Extended Data Figure 7-1 — Inhibition experiments following the test of two concentrations and measuring ATP levels until 120 min after axotomy. a, A schematic illustration of inhibition experiments. The following inhibitors was used; 2-DG, MSDC-1060, and 4-CIN. The control was set as 0.1% DMSO dissolved in saline. Bilateral sciatic nerves were exposed, and transection side as right, sham side as left. Immediately after the transection of right sciatic nerve, bilateral sciatic nerves were covered with a gelatin sponge soaked with inhibitors. One day after the surgical procedure, ATP levels and histology were evaluated in 3 mm distal from the sectional end. b, Energy metabolic interactions between Schwann cells and axon, and the working point of the inhibitors. Monocarboxylate produced by the Schwann cell glycolytic system is transported to axons through MCTs. Pyruvate, the end product of the glycolytic system, is transported to the mitochondrial matrix through MPC and metabolized in the TCA cycle and electron transport chain. 2-DG is a glycolysis inhibitor, MSDC-0160 is the MPC inhibitor, and 4-CIN is the comprehensive MCTs inhibitor. c, Representative intracellular distal nerve stump ATP levels for each inhibitor compared to the DMSO group, before (pre) and 15, 30, 45, 60, 75, 90, 105, and 120 min after axotomy. Scale bar, * 1mm. P, proximal. D, distal. d, ATP reduction response for two different concentrations of each inhibitor, compared to the DMSO group. Two concentrations of several inhibitors were preliminary tested. *p < 0.05, two-way ANOVA followed by the Tukey’s post hoc test. The ROI for measuring ATP levels was set at 3 mm distal to the sectional end, with a 500 × 500-μm square (Extended Data Fig. 7-2). A higher concentration (150 mm 2DG, 100 μm MSDC-0160, and 10 mm 4-CIN) was applied for experiment in all inhibitors. The OFP/GFP ratios ranged from 1.5 to 3.0. Error bars indicate SD. *p < 0.05, one-way ANOVA followed by Tukey’s post hoc test. Download Figure 7-1, TIF file. [file enu-eN-CFN-0353-22-s03.tif]

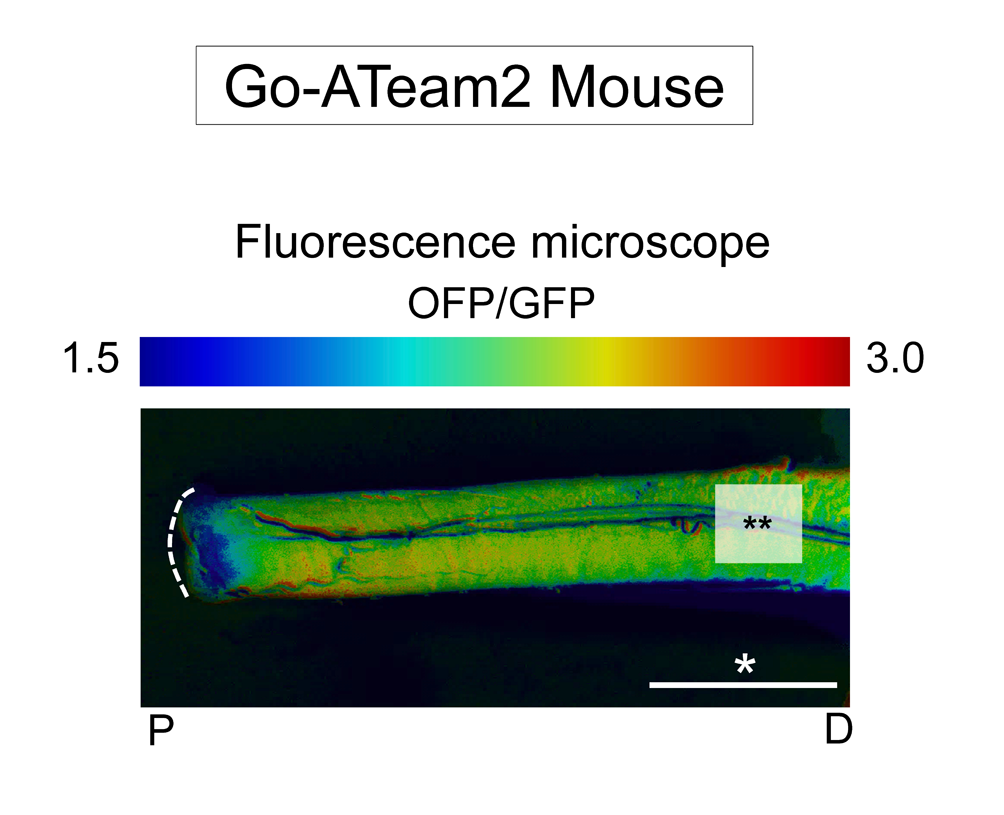

Supplement: Extended Data Figure 7-2 — Experimental system for ATP levels with fluorescence microscopy in mice. A schematic illustration of whole nerve ATP levels measurement for GO-ATeam2 mouse. All of fluorescence emission in the GO-ATeam2 probe was captured with fluorescence microscopy. ATP levels were measured in each ROIs (** 500-μm square), 3 mm distal from the sectional end (broken line). OFP/GFP ratio ranges were from 1.5 to 3.0. The histological analysis was performed 3 mm distal from the sectional end. Scale bar, * 1 mm;** 500-μm square ROI. *** 500-μm square ROI. P, proximal. D, distal. Download Figure 7-2, TIF file. [file enu-eN-CFN-0353-22-s04.tif]
