# Supplementary material for: Glycolytic System in Axons Supplement Decreased ATP Levels after Axotomy of the Peripheral Nerve
Source: eNeuro. 2023 Mar 17;10(3):ENEURO.0353-22.2023. doi: 10.1523/ENEURO.0353-22.2023 (PMC10035771; doi:10.1523/ENEURO.0353-22.2023)
Supplement: Extended Data Table 7-1 — The summary of the inhibition experiments. The downward arrow means a decrease in the value of each variable relative to the horizontal arrow, whereas upward arrow means an increase. The more numbers of arrows, the greater the change in value. 2-DG, 2-deoxyglucose; 4-CIN, a-cyano-4-hydroxycinnamic acid; MCT-1, monocarboxylate transporters 1; MSDC-0160, mitochondrial pyruvate carrier (MPC) inhibitors. Download Table 7-1, DOCX file. [file enu-eN-CFN-0353-22-s05.docx]

Table 7-1. The summary of the inhibition experiments.

Sham side

| Evaluated function | Variable | DMSO | 2-DG | MSDC-0160 | 4-CIN |
| --- | --- | --- | --- | --- | --- |
| Energy currency | ATP | → | ↓ | ↓ | → |
| Monocarboxylate transport | MCT-1 positive schwann cell | → | ↑ | ↑ | ↑ |
| Mitochondrial atypicality | Axon | → | ↓↓ | ↓ | → |
|  | Schwann cell | → | ↓ | ↓ | ↓ |
| WD progression | Myelinated axon | → | ↓ | ↓ | ↓ |
|  | G-ratio | → | ↓ | → | → |

Transection side

| Evaluated function | Variable | DMSO | 2-DG | MSDC-0160 | 4-CIN |
| --- | --- | --- | --- | --- | --- |
| Energy currency | ATP | ↓ | ↓↓ | ↓ | ↓↓ |
| Monocarboxylate transport | MCT-1 positive schwann cell | ↑ | ↑↑ | ↑↑ | ↑↑ |
| Mitochondrial atypicality | Axon | ↓↓ | ↓↓ | ↓↓ | ↓↓↓ |
|  | Schwann cell | ↓ | ↓ | ↓↓ | ↓↓↓ |
| WD progression | Myelinated axon | ↓↓ | ↓↓↓ | ↓↓ | ↓↓↓↓ |
|  | G-ratio | ↓ | ↓↓ | ↓ | ↓↓↓ |
